# Objective measurements of skinfold thickness with a caliper show a significant relationship to total body fat percentage in dogs

**DOI:** 10.3389/fvets.2025.1656855

**Published:** 2025-09-12

**Authors:** Josefin Söder, Ida Eskol Svenningsen, Julie Baltzer Larsen, Mette Hedelund Rasmussen, Fintan J. McEvoy, Kathrine Stenberg, Anna Bergh, Charlotte Reinhard Bjørnvad

**Affiliations:** ^1^Department of Clinical Sciences, Faculty of Veterinary Medicine and Animal Science, Swedish University of Agricultural Sciences, Uppsala, Sweden; ^2^Department of Veterinary Clinical Sciences, Faculty of Health and Medical Sciences, University of Copenhagen, Frederiksberg, Denmark

**Keywords:** body condition score, BCS, bodyweight, clinical assessment, dual-energy X-ray absorptiometry, DEXA, overweight, reliability

## Abstract

**Objective:**

New clinical evaluation methods for estimation of total body fat percentage (BF%) in dogs are needed. The methods should be objective and reliable for accurate assessment of body composition status and to improve prevention and treatment of obesity. The aims of the study were therefore to investigate the intra- and inter-observer reliability of objective measurements of skinfold thickness with a caliper and to explore the relationship of skinfold thickness to dual-energy X-ray absorptiometry (DEXA) BF% in dogs.

**Methods:**

Twenty-three carcasses of dogs euthanatized for reasons unrelated to the study were evaluated for body condition score (BCS), bodyweight, skinfold thickness, and DEXA BF%. The results from the latter were taken as gold standard for BF% measurement. The cohort consisted of 14 different breeds, aged ≥1 year. Objective measurements of skinfold thickness were collected in triplicate by two blinded observers at the locations of the “dorsal neck,” “axillar rib,” and “lumbar back.” Statistical analyses explored intra-class correlation coefficients (ICCs) and relationships by linear models and generalized additive models (GAMs).

**Results:**

The dogs had a BCS of 2–9/9, a bodyweight of 2–52 kilograms, and a BF% of 6.4–74.7 percent. Objective measurements of skinfold thickness showed high intra- (range 0.991–0.993) and inter- (range 0.937–0.977) observer reliability at all locations. The skinfold thickness of the “axillar rib” in interaction with bodyweight within a spline (*p* = 0.0001), plus the “dorsal neck” as a linear variable (*p* = 0.0004), explained 73.4% of the variation in DEXA BF%. The BF% of small-sized dogs were over- and under-predicted by the prediction equation to a larger extent than for dogs of larger sizes. Due to the interaction with bodyweight, a slight variation in the low measurement values of the skinfold thickness corresponded to a large variation in DEXA BF%.

**Conclusion:**

Objective measurements of skinfold thickness could be assessed with high reliability with a caliper and showed a significant non-linear relationship to DEXA BF%. Longitudinal clinical studies with larger cohorts of small-, medium-, and large-sized dogs of different breeds and BCS are warranted, to evaluate the caliper device for its potential to follow changes of BF% over time. Objective measurements of skinfold thickness may in the future be practically implemented in nutritional assessments of dogs.

## Introduction

The prevalence of overweight is reaching rates of up to 40% in the companion dog populations in Sweden and Denmark (1–3). This is a great concern as an overweight condition is known to decrease quality of life, increase risk of co-morbidities, and decrease life expectancy (4–7). Development of overweight is multifactorial, but a key factor is probably the difficulty in identifying the developing overweight status in the individual dog (1). Being able to accurately assess the body composition status of a dog is essential for both early detection of excess body fat and for correct planning and performance of effective weight loss programs. Assessment of fat mass in dogs is often clinically performed using a body condition score (BCS) system, a well-established but semi-subjective method (8–10), in combination with the recording of bodyweight. Bodyweight is useful for monitoring weight development over time but does not provide information on the fat-to-muscle ratio (11). The body fat index (BFI) system is another method to assess fat mass in dogs by morphometric measurements (12). The BFI system is, however, adapted for overweight to obese dogs and not for underweight to normal weight dogs. The BCS system, on the other hand, does not always perform well in dogs of different body conformation, resulting in breed-related variations in body fat percentage in dogs assessed to have the same BCS score (13).

Weight-loss interventions in overweight dogs include nutritional intervention (14) and/or recommendations of increased physical activity (11, 15). During such interventions, the dog’s body composition is expected to change, with the aim of reducing fat mass and maintaining muscle mass (11, 15) but with the current BCS system, changes in fat and muscle mass can be difficult to capture. There is consequently a need for development of new clinical methods enabling differentiation between fat and muscle mass, to improve diagnosis, treatment, and follow-up of a large number of overweight dogs. Preferably, the new methods should be validated and “objective” (16), i.e., independent of individual perceptions or biases.

The gold-standard method for assessment of total body fat percentage (BF%) in dogs (17) as in people (18) is dual-energy X-ray absorptiometry (DEXA). The method is considered the second best after carcass analysis (17) and is precise (19, 20), but costly, and requires anesthesia to be performed on dogs (10). Various canine studies have established a linear relationship between the clinical BCS system and BF% evaluated by DEXA (8, 9, 19). Laflamme (8) developed the 9-point BCS system on a cohort of three breeds plus mixed breeds. Thereafter, Mawby et al. (9) validated the system on a cohort of 10 breeds. Even though several breeds were represented, these studies showed excellent correlations between BCS and DEXA BF% (8, 9). On the contrary, Jeusette et al. (13) showed significantly different DEXA BF% in dogs that were evaluated to be normal to slightly overweight (mean BCS 5.7/9), when genetically different breeds were investigated. The question has therefore been raised, whether clinical evaluation methods of BF%, such as the BCS system, should be adapted to fit genetically diverse dog breeds (9, 13).

New objective methods, such as measurements of skinfold thickness with a caliper, could be important complementing methods to the clinical BCS system for a more precise assessment of BF% in dogs. Although objective, the complementing method still needs to be reliable. Ideal measures demonstrate high intra- and inter-observer reliability when compared within and between observers (16). In people, objective measurements of skinfold thickness with a caliper have been described as reliable and suitable for clinical settings (21, 22). Similar to other anthropometric measures, the reliability of caliper measurements may be affected by factors such as experience of the observer (23, 24), localization of the measurement site (25, 26), performance of the device (27), and the body composition of the subject (28, 29). To our knowledge, objective measurements of skinfold thickness with a caliper in dogs have not previously been evaluated for reliability.

Objective measurements of skinfold thickness with a caliper, as a clinical method for prediction of BF%, have been described within human medicine since the 1960s (30, 31). When used in clinical settings at population or at individual level, selection of a suitable equation for the prediction of BF% from the caliper measurements is essential (18, 21, 32). In human medicine, various multivariable regression models based on different measurement locations have been described for direct prediction of BF% (30, 33–35). Since the millennium, most prediction equations have been developed and/or validated against DEXA BF% (18, 32, 36, 37). The relationships between measurements of skinfold thickness and BF% in people have been described as linear (33, 35, 36), quadratic (33), or non-linear (38). Anthropometric-based predictive equations for estimation of BF% have proven robust in, e.g., athletes and children (18, 28), and its usefulness in veterinary medicine merits investigation. In veterinary medicine, there is only one previous canine study which has described objective measurements of skinfold thickness with a caliper at the location of the lumbar back and showed a significant correlation to BCS (39). The relationship between objective measurements of skinfold thickness with a caliper and BF% evaluated by DEXA in dogs has, to the authors’ knowledge, not been described previously. In the current study, we therefore performed objective measurements of skinfold thickness on three anatomical locations with a caliper in underweight to obese dogs, and the measurements were compared to DEXA BF%. The aims of the study were to investigate intra- and inter-observer reliability and to explore the relationship of skinfold thickness to DEXA BF% in dogs.

## Materials and methods

### Ethics approval and owner consent

Ethics approval (Acceptance No. 2024-24) was authorized by the Local Ethical and Administrative Committee at the Department of Veterinary Clinical Sciences, University of Copenhagen, Denmark. All dog owners signed informed consent allowing their dog to be used for research and educational purposes following euthanasia.

### Recruitment of study population

The study population consisted of a cross-sectional cohort of newly euthanized dogs at the University Hospital for Companion Animals, University of Copenhagen, Denmark. The sample was a convenience sample of dogs presenting for euthanasia, for reasons unrelated to the study. The inclusion criteria were that dog owners had signed consent for their dog to be donated for research and educational purposes following euthanasia. Dogs of any breed and size, ≥1 year of age were included in data collection during a 2-month period (2nd of September 2024 until the 8th of November 2024). Dogs were excluded if they were severely dehydrated (>10%), had fluid accumulation, traumatic injuries, tumors ≥5 cm in diameter at any site, a tumor of any size located at a location for measurements of skinfold thickness, a diagnosis of hyperadrenocorticism, or had received systemic steroid treatment prior to euthanasia.

### Clinical data collection

All dogs were euthanized according to the wish of the owner, following the standard hospital protocol by an authorized veterinarian. Immediately following euthanasia, the dogs were placed on a table in ventral recumbency with thoracic limbs pulled cranially and pelvic limbs pulled caudally. Dogs were only included if it was logistically possible to perform all measurements within 2 h following euthanasia, to minimize effects of water evaporation and the onset of rigor mortis. Data were collected in the following order: bodyweight, BCS, subjective skinfold thickness by palpation with fingers only, objective measurements of skinfold thickness with a caliper, and DEXA scanning. The dogs were evaluated by two veterinary Master students, and the starting order for evaluation by the students was randomized for each dog. The two students had received training in clinical BCS assessment as well as in performing the measurements of skinfold thickness before the data collection started. The two students will from hereon be called “observers.”

#### Bodyweight and body condition score assessment

The dog’s bodyweight (Kruuse scale 250, Kruuse, Langeskov, Denmark) was noted by hospital staff prior to euthanasia. The BCS was individually assessed by the two observers, and afterward, an agreement was reached on a score which was the BCS score recorded as data. The 9-point BCS scale developed by Laflamme (8) was used for body condition assessments, with the exception that the abdominal tuck was not possible to assess accurately postmortem. The BCS assessment was performed primarily by palpating the ribs, and the waist was assessed visually (viewed from above) and by hand palpation. For the assessment of the waist, the dog was elevated from the table by lifting the pelvis and sternum from the ventral side, to not influence the waistline. The lifting was performed either by both observers simultaneously or by one of the observers, depending on the bodyweight of the dog.

#### Subjective and objective measurements of skinfold thickness

**Figure 1:**
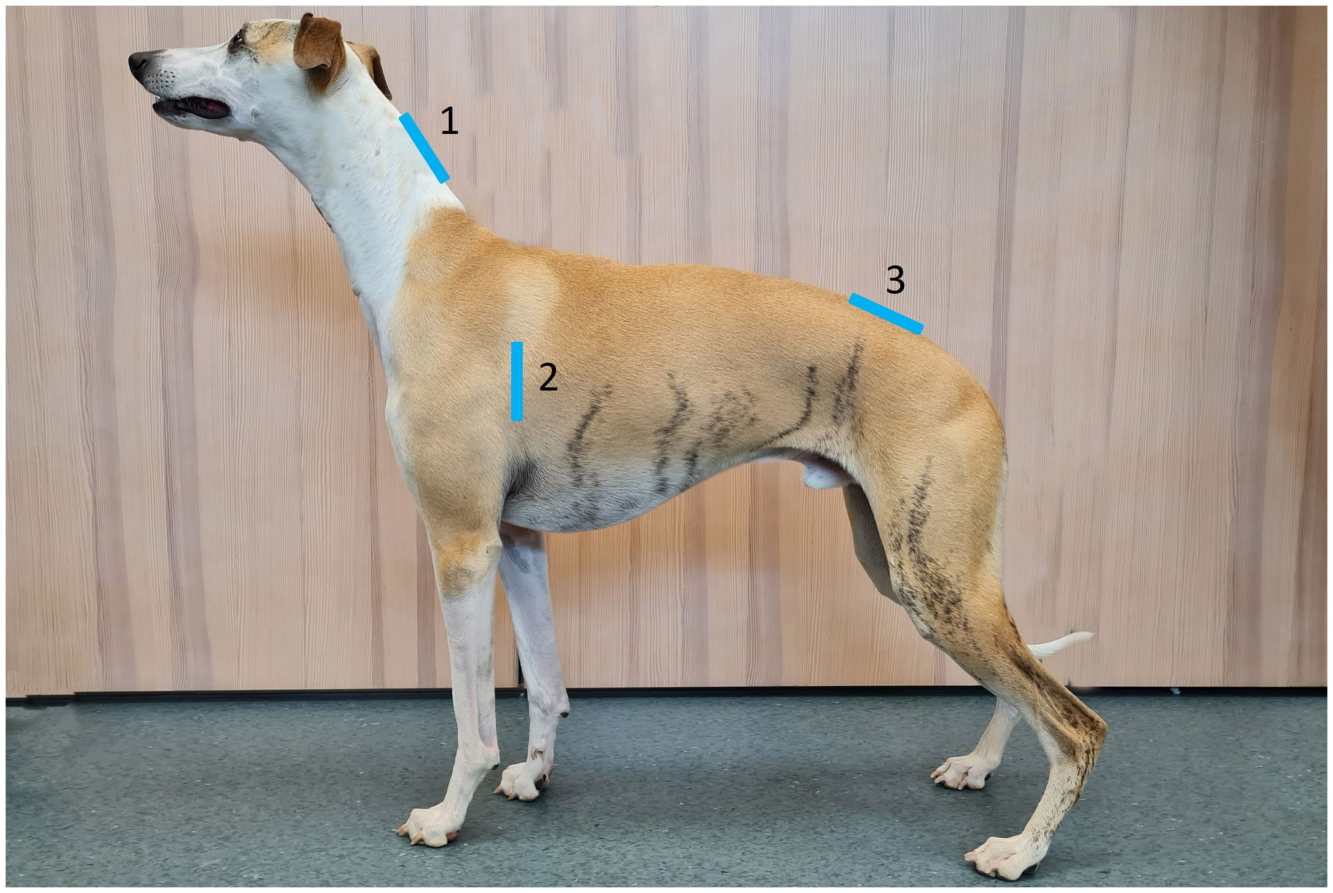
Anatomical locations for subjective and objective measurements of skinfold thickness. The location at the dorsal neck (1) was measured at half of the distance from the midpoint in between the cranio-proximal part of the scapular bones to the crista nuchae on the skull. The location of the axillar rib (2) was measured at half of the distance between the spinal processes of the vertebrae and the sternal bone, just caudal to the axilla and in parallel to the 4th–5th rib. The location at the lumbar back (3) was measured just cranially to the midpoint in between the cranio-proximal part of the wings of ilium on the pelvic bone. The blue bars represent the bases of the skinfolds. Photo and editing: Josefin Söder.

The height of the lifted skinfold was measured by a tape measure and recorded in the nearest 0.5 centimeters (cm), aiming for a skinfold height of 2–5 cm in all included dogs. The skinfold of the dorsal neck and lumbar back was picked up parallel to the sagittal plane of the dog, and the skinfold of the axillar rib was picked up in parallel to the 4th–5th rib. At the locations of the dorsal neck and lumbar back, the caliper was held in vertical position, and at the location of the axillar rib, the caliper was held in horizontal position. Subjective and objective measurements of skinfold thickness were performed at the locations of the dorsal neck, axillar rib, and lumbar back by both observers. The location at the dorsal neck was identified by measuring half of the distance from the midpoint in between the cranio-proximal part of the scapular bones, to the crista nuchae on the skull, by a tape measure. The location of the axillar rib was identified by measuring half of the distance between the spinal processes of the thoracic vertebrae and the sternal bone by a tape measure, just caudal to the axillary fold in the axilla. After that the height of the location had been identified with the tape measure, the ribs were counted, and the measurement was performed parallel to the 4th–5th rib on all dogs. The location at the lumbar back was identified by palpating the midpoint in between the cranio-proximal part of the wings of ilium on the pelvic bone, and the skinfold was picked up just cranial to this midpoint. Only one side of the thorax was evaluated for the axillar rib, and the lateralization (right vs. left) was randomized so that half of the dogs were evaluated for each side. The measurement locations of the dorsal neck, axillar rib, and the lumbar back were chosen to represent the locations for evaluation of the subcutaneous fat layer and/or fat deposits in the 9-point BCS system (8). The subjective and objective measurements of skinfold thickness were performed on the same three locations, which are marked in Figure 1.

##### Subjective measurements of skinfold thickness

A subjective skinfold thickness was assessed by pinching the skin between the thumb and the index finger with one hand. The subjective skinfold thickness was assessed by subjective estimation of the distance between the thumb and index finger at the base of the skinfold. The observers used the same (dominant) hand in all subjective assessments, and the estimated value of the skinfold thickness was recorded with the other observer blinded to the result. Due to the nature of the procedure, the observer performing the assessment could not be blinded to the value, and the subjective assessment was therefore performed in one measurement replicate only. Both observers estimated the subjective skinfold thickness independently and registered the values, and thereafter, objective measurements of skinfold thickness with a caliper were performed.

##### Objective measurements of skinfold thickness

The objective measurements of skinfold thickness were performed in a blinded procedure, where one observer placed the caliper device while the other observer recorded and registered the value, so that the procedure was independent of individual perceptions or biases. The objective measurements of skinfold thickness with the caliper were collected in triplicate to the nearest 0.1 millimeter (mm) by each of the two observers at each location using a “Harpenden Skinfold Caliper” (Baty International Ltd., West Sussex, United Kingdom) with 10 grams/mm^2^ constant spring pressure. The caliper was replaced for each replicate. The skinfold was held during the measurements of all triplicates at each location. However, when reading the value from the display, the skinfold was temporarily released. Before the measurements on each dog, the caliper was inspected and the display calibrated to start on zero if needed.

#### Dual-energy X-ray absorptiometry scanning procedure

The choice of DEXA as the reference method was made to enable exploration of a possible relationship of objective measurements of skinfold thickness and BF%, a value that is obtained by the DEXA technique. The technicians at the radiology department performed the DEXA scans. The procedure was blinded to the observers in that the results of the DEXA scans were not known to the observers at the time of BCS assessment or measurements of skinfold thickness. All DEXA scans were obtained with the same DEXA scanner (GE Health Care United States, Lunar Prodigy, Illinois, United States), which was calibrated twice a week by the technicians in the radiology department using a calibration phantom. The dogs were placed in ventro-dorsal recumbency with the thoracic limbs pulled cranially and the pelvic limbs caudally. A full body scan was performed on all dogs, according to the procedure described in Supplementary File 1. The DEXA scanning replicates for each dog were obtained in duplicate, triplicate, or quadruplicate. The dogs were not repositioned between scans.

##### Dual-energy X-ray absorptiometry data extraction

The DEXA results were extracted using the software enCORE (version 1360.2010.1214.33), where the placement of the “Regions of Interest” (ROI) was performed according to the user instructions for people and small animals (40) and is described in Supplementary File 1. The DEXA scanning replicates entailed a repeated run of the DEXA scanner, so that new data were acquired, re-placing the ROIs was performed as was the re-analysis by the software.

### Data processing and statistical analyses

Microsoft Excel, GraphPad Prism (GraphPad Prism 5.0 San Diego, CA), and RStudio (2023.12.0 + 369 Posit Software, PBC) were used for data processing, statistical analyses, and graphical presentation. D’Agostino and Pearson omnibus normality tests were used for evaluation of normal distribution of caliper data. Only the caliper measurements of the dorsal neck were normally distributed, and comparison of values between and within observers was therefore performed with non-parametric analysis techniques for all measurements. The results are therefore presented as median and range. The precision of objective measurements of skinfold thickness is presented as the mean standard error of mean (mean SEM) of the triplicate. Means of the triplicate were used in the analyses of objective measurements of skinfold thickness between observers, and the means of the triplicate were also compared to the subjective skinfold assessments, within observer. The mean of the total body fat percentages (BF%) from the DEXA scan replicates (in duplicate, triplicate, or quadruplicate) was calculated for each dog, and the precision of the DEXA scan replicates was calculated with the coefficient of variation (CV) per dog. In the regression analyses, the means of the objective measurements of skinfold thickness with the caliper from both observers were used as data, as well as the mean BF% from the DEXA scan replicates. The threshold for statistical significance was set to *p* < 0.05 for all analyses.

#### Paired analyses

Wilcoxon signed rank tests were used for comparisons of objective measurements of skinfold thickness with the caliper between observers at each location. Wilcoxon signed rank tests were also used for comparisons of objective measurements of skinfold thickness and subjective assessment of skinfold thickness, within observers, at each location.

#### Calculations of intra- and inter-observer reliability of objective measurements of skinfold thickness with the caliper

The libraries “tidyverse” (41) and “ICC” (42) in RStudio were used for calculation of intraclass correlation coefficients (ICCs) with 95% confidence interval (CI) for the objective measurements of skinfold thickness with the caliper. Reliability within observer (intra-observer reliability) was calculated from the individual measurements in triplicate, for each observer, at each location (dorsal neck, axillar rib, and lumbar back). Reliability between observers (inter-observer reliability) was calculated from the means of the measurements in triplicate, between observer 1 and observer 2, at each location. The interpretations of all ICC were based on previously established levels of reliability: high reliability, 0.90–0.99; good reliability, 0.80–0.89; fair reliability, 0.70–0.79; moderate reliability, 0.69–0.59; and poor reliability, <0.59 (43).

#### Regression analyses

Linear regression analyses were used to determine linear relationships between objective measurements of skinfold thickness with the caliper and BF% evaluated by DEXA, per respective measurement location. Analyses were performed in RStudio with the library “tidyverse” (41) using linear models (lm) with the objective measurements of the dorsal neck, the axillar rib, and lumbar back as *x*-variables and DEXA BF% as *y*-variable, in three separate analyses.

A Spearman correlation matrix table of the numeric variables (dorsal neck, axillar rib, lumbar back, age, and bodyweight) was created to explore possible multicollinearity or relationships among variables. The library “mgcv” (44) in RStudio was used for exploring linear and non-linear relationship and combinations of those using generalized additive models (GAMs). The BF% evaluated by DEXA was set as the *y*-variable in all models, and the objective measurements of skinfold thickness of the dorsal neck, axillar rib, and lumbar back were set as *x*-variables. Dog intrinsic factors such as bodyweight, age, sex, and neutering status were also added to the most significant model as *x*-variables, where sex, size, and neutering status of dogs were defined as categorical variables. The models were adjusted with forward and backward selection of variables and with application of interactions (by) and spline effects (s) so that the highest deviance possible of the DEXA BF% was explained, without inflated standard errors and with the assumptions met for the error term. Equations that presented the following criteria were selected in the process of fitting a final model: a higher value of the deviance explained (DE), lower standard errors (SE), a lower Akaike information criterion (AIC), and fewer independent variables included. The measurement locations were tested one by one, as well as in different combinations with each other. In the final GAM model used for prediction of BF%, the dogs were color-coded according to three bodyweight groups: small size (S) <10 kg, medium size (M) 10–25 kg, and large/giant size (LG) >25 kg, but the size of dogs was not included as a variable in the model.

On the final GAM model, leave-one-out cross-validation (LOOCV) was performed for estimation of performance metrics. The LOOCV was performed accordingly: the loop ran once for each dog (*n* = 23). Each iteration of the loop left one dog out for testing and used the remaining 22 dogs for training. The code fitted a new model on the 22 “training dogs” and predicted the left-out dog. After that the 23 loops were completed, the root mean square error (RMSE), the mean bias and its 95% CI, and the slope of the predicted versus the observed values on BF% were calculated across all cross-validation loops.

## Results

Twenty-three dogs met inclusion criteria and were included in the data collection. All dogs were evaluated for subjective and objective measurements of skinfold thickness, and in total, 396 caliper measurements were performed. DEXA scanning of dogs was performed in duplicate on 10 dogs, in triplicate on 11 dogs, and in quadruplicate on two dogs. Two of the DEXA scanning replicates were excluded because the dogs were off center or crooked in the scans.

In the assessment of two dogs, only one of the observers was present. During these occasions, an assistant read and recorded the values from the caliper device, and the observer thus had the same blinded routine as with the other dogs. The order of the original data collection was changed in five of the 23 dogs, which was due to logistic priorities at the diagnostic imaging department. In those five dogs, BCS was evaluated after the DEXA scanning, instead of before. The data from the DEXA scanning replicates were extracted from the machine’s local database on 7 March 2025, after which all 23 dogs were evaluated. The observers were therefore blinded to the results on DEXA BF% during all BCS assessments and during all measurements of skinfold thickness, regardless of data collection order.

### Descriptive data of the dog cohort

The dog cohort displayed a wide range in age, bodyweight, and BCS (Table 1). The gender distribution was quite even, as was the neuter status in male and female dogs (Table 1). The cohort consisted of 14 different breeds, including mixed breed. The breeds were Mixed breed, (6; of which 3 = small; 2 = medium; and 1 = large/giant), Chihuahua (4), Poodle (2), Bichon Havanais (1), Border Collie (1), Cane Corso (1), Coton de Tulear (1), German Shepherd (1), Golden Retriever (1), Old English Bulldog (1), Perro de Agua Español (1), Rottweiler (1), Staffordshire Bull Terrier (1), and West Highland White Terrier (1).

**Table 1:** Descriptive data of the dog cohort composed of 23 privately owned newly euthanized dogs.

	Dog cohort (*n* = 23)
Parameter	Median (range)
Age (years)	3 (1–15)
Bodyweight (kg)	19.5 (2–52)
BCS (scale 1–9)	5 (2–9)

BCS (scale 1–9)	Number of dogs and their size S/M/LG
2 (underweight)	1S
3 (slight underweight)	1S/1M
4 (normal weight)	3S/2M/2LG
5 (normal weight)	2S/2M/2LG
6 (slight overweight)	2S/1M
7 (overweight)	2LG
8 (obese)	1M
9 (obese)	1S

Sex	Number of dogs
Male (of which neutered)	13 (3)
Female (of which spayed)	10 (1)

BCS, body condition score; kg, kilogram; S, small-sized dog <10 kg; M, medium-sized dog 10–25 kg; LG, large- and giant-sized dogs >25 kg; SD, standard deviation.

### Subjective and objective measurements of skinfold thickness

Subjective assessment of skinfold thickness by palpation with fingers only and objective measurements of skinfold thickness with a caliper did not differ within any observer, at any location, at cohort level (Table 2). For individual dogs, however, the difference between the two methods within observer, within one location, was up to 6 millimeters (mm) (Supplementary File 2). Objective measurements of skinfold thickness with the caliper did not differ between observers, but the location of the axillar rib showed a numerically higher difference in median skinfold thickness than the other locations (Table 2). Precision (mean SEM) of the objective measurements of skinfold thickness in triplicate was approximately 0.1–0.2 mm for both observers. The mean ± SD height of the lifted skinfolds was 3.8 ± 0.5 cm for the dorsal neck, 3.0 ± 1.2 cm for the axillar rib, and 3.0 ± 1.0 cm for the lumbar back (Supplementary File 2).

**Table 2:** Comparisons between observers of objective measurements of skinfold thickness and comparisons between subjective and objective measurements of skinfold thickness within observer, in 21 privately owned newly euthanized dogs.

	Within observer 1				Within observer 2				Between observer 1 and 2
Location	Subjective skinfold thickness (mm)	Objective skinfold thickness (mm)	Difference of subjective vs. objective	Precision of objective measurements (mm)	Subjective skinfold thickness (mm)	Objective skinfold thickness (mm)	Difference of subjective vs. objective	Precision of objective measurements (mm)	Difference of objective measurements
	Median (range)	Median (range)	*p*-value	Mean SEM ± SD	Median (range)	Median (range)	*p*-value	Mean SEM ± SD	*p*-value
Dorsal neck	7.0 (2.0–15.0)	7.4 (2.8–18.2)	0.72	0.18 ± 0.12	6.0 (2.0–15.0)	7.3 (2.6–18.3)	0.30	0.18 ± 0.14	0.93
Axillar rib	6.0 (1.5–18.0)	5.9 (1.9–15.4)	0.92	0.16 ± 0.11	5.0 (1.0–15.0)	5.1 (1.5–15.5)	0.67	0.15 ± 0.10	0.39
Lumbar back	7.0 (2.0–18.0)	6.5 (2.2–17.5)	0.65	0.15 ± 0.11	6.0 (1.0–15.0)	6.4 (2.4–15.5)	0.50	0.13 ± 0.11	0.79

SEM, standard error of mean. The table shows data from the 21 dogs that were evaluated by both observers. Mann–Whitney tests were used for comparison of objective measurements of skinfold thickness between observers, at each location. Mann–Whitney tests were also used for comparison of subjective and objective measurements of skinfold thickness within observer, at each location. A *p*-value of <0.05 was considered significant.

### Intra- and inter-observer reliability of objective measurements of skinfold thickness

Objective measurements of skinfold thickness with the caliper showed overall high intra- (range 0.991–0.993) and inter- (range 0.937–0.977) observer reliability at all anatomical locations according to the calculated ICC (Table 3). Intra-observer reliability was high and comparable between observers. Inter-observer reliability was high for all anatomical locations, but the location of the axillar rib showed a numerically, slightly lower inter-observer reliability (Table 3).

**Table 3:** Intra- and inter-observer reliability (ICC) of objective measurements of skinfold thickness in 21 to 23 privately owned newly euthanized dogs.

Location	Intra ICC (95% CI)		Inter ICC (95% CI)
	Observer 1	Observer 2	
Dorsal neck	0.992 (0.984–0.997)	0.992 (0.983–0.996)	0.977 (0.945–0.990)
Axillar rib	0.991 (0.981–0.996)	0.992 (0.984–0.996)	0.937 (0.856–0.973)
Lumbar back	0.993 (0.985–0.997)	0.993 (0.986–0.997)	0.956 (0.899–0.981)

ICC, intraclass correlation coefficient; CI, confidence interval. All individual measurements of skinfold thickness in triplicate were included in the analyses of intra-observer reliability, and the mean of the triplicate measurements was used for analyses of inter-observer reliability. Analyses were performed with a 95% confidence interval on data from 23 dogs for intra-observer reliability of observer 2, on 21 dogs for observer 1, and on 21 dogs for analyses of inter-observer reliability.

### Dual-energy X-ray absorptiometry results

Total body fat percentage (BF%) evaluated by DEXA for the dog cohort was median 41.3% (range 6.4–74.7). The coefficient of variation (CV) of the DEXA scan replicates per individual dogs was median 1.0% (range 0–4.3) in the dog cohort. BF% evaluated by DEXA, as well as CV of the DEXA scan replicates for underweight, normal weight, overweight, and obese groups of dogs are shown in Table 4.

**Table 4:** Total body fat percentage (BF%) evaluated by dual-energy X-ray absorptiometry (DEXA) and coefficient of variation (CV%) of DEXA replicates in 23 privately owned newly euthanized dogs.

	Dog cohort (n = 23)	
BCS (scale 1–9)	DEXA BF%	CV% of DEXA replicates
	Median (range) (%)	Median (range) (%)
2–3 (underweight, *n* = 3)	11.3 (6.4–34.9)	2.5 (1.0–2.7)
4–5 (normal weight, *n* = 13)	33.5 (7.3–48.3)	1.4 (0–4.2)
6–7 (overweight, *n* = 5)	43.2 (31.0–62.3)	0.9 (0.6–1.4)
8–9 (obese, *n* = 2)	69.5 (64.2–74.7)	0.2 (0.21–0.22)

BCS, body condition score; BF%, total body fat percentage; CV%, coefficient of variation; DEXA, dual-energy X-ray absorptiometry. The CV% was calculated from the DEXA scanning replicates per individual dogs. DEXA BF% and its corresponding CV% are here presented as median and range in underweight, normal weight, overweight, and obese groups of dogs. The CV of the underweight group of dogs is numerically higher than for the other groups, but the CV might potentially be inflated because of the low number of underweight dogs included and should be interpreted with caution.

### Relationship of measurements of objective skinfold thickness with a caliper and total body fat percentage

The mean BF% from the DEXA scanning replicates from each individual dog was set as *y*-variable in the exploration of relationships between objective measurements of skinfold thickness and BF%. The script for all statistical analyses is available in Supplementary File 3.

#### Linear relationships

**Figure 2:**
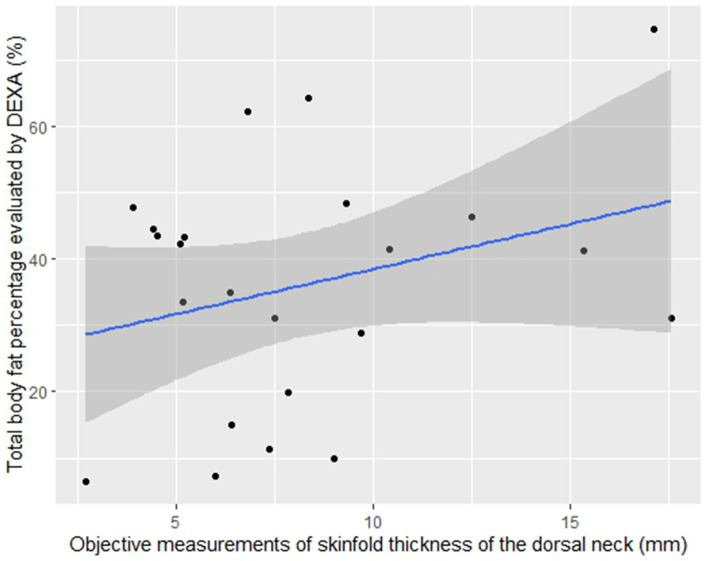
Scatter plot of objective measurements of skinfold thickness of the dorsal neck and DEXA BF%. Objective measurements of skinfold thickness in millimeter (mm) are set as *x*-variable (scale of *x*-axis: 2.5–17.5 mm) and total body fat percentage (BF%) is set as *y*-variable (scale of *y*-axis: 0–80%). BF% was evaluated by dual-energy X-ray absorptiometry (DEXA). The line shows the fitted equation (*y* = 24.97 + 1.35*x*) of the linear model where each dot represents the mean value from the objective measurements of skinfold thickness in triplicate and the mean value from the DEXA replicates from all dogs (*n* = 23). The scatter plot shows a non-significant linear relationship (*p* = 0.16).

All anatomical locations showed a non-significant linear relationship of objective measurements of skinfold thickness and BF% (Table 5) when analyzed separately. The location of the dorsal neck showed the highest R^2^; however, the relationship to DEXA BF% was still not significant (*p* = 0.16) (Figure 2).

**Table 5:** Linear relationships of objective measurements of skinfold thickness and total body fat percentage (BF%) evaluated by dual-energy X-ray absorptiometry (DEXA) in 23 privately owned newly euthanized dogs.

Statistical model	Coefficients of linear variables			Explained variation	
Simple linear models (lm)	Estimate skinfolds	SE skinfolds	*p*-value skinfolds	*R*^2^ Adj.	*R* ^2^
lm (BF% ~ dorsal neck)	1.35	0.94	0.16	0.05	0.09
lm (BF% ~ axillar rib)	0.97	1.15	0.41	−0.01	0.03
lm (BF% ~ lumbar back)	1.12	1.12	0.33	9.5 × 10^−6^	0.05

lm, linear model; BF%, total body fat percentage; SE, standard error; *R*^2^ Adj., coefficient of determination adjusted; *R*^2^, coefficient of determination. Data were analyzed by linear models with objective measurements of skinfold thickness as *x*-variables and total body fat percentage (BF%) as *y*-variable.

#### Non-linear relationships

Addition of spline effects to the objective measurements of skinfold thicknesses to the separate locations did not create significant relationships to DEXA BF% (*p* ≥ 0.17), although a “spline” allows the relationship to take any shape. However, after the addition of an interaction with bodyweight for the separate locations for objective measurements of skinfold thickness within the spline effects, the relationships became significant for all anatomical locations (Table 6).

**Table 6:** Non-linear relationships of objective measurements of skinfold thickness and total body fat percentage (BF%) evaluated by dual-energy X-ray absorptiometry (DEXA) in 23 privately owned newly euthanized dogs.

Statistical model	Smooth terms (splines)		Explained variation	
Splines (s) and interactions (by) in generalized additive models (GAMs)	edf splines	*p*-value splines	*R*^2^ Adj.	DE%
gam (BF% ~ s (dorsal neck, by = bodyweight))	3.02	0.01	0.38	46.5
gam (BF% ~ s (axillar rib, by = bodyweight))	3.16	0.02	0.36	45.3
gam (BF% ~ s (lumbar back, by = bodyweight))	3.12	0.01	0.39	47.2

s, spline; by, “in interaction with”; gam, generalized additive model; edf, estimated degrees of freedom, i.e., the model effect; *R*^2^ Adj., coefficient of determination adjusted; BF%, total body fat percentage; DE, deviance explained, i.e., the explained variation of total BF%. Data were analyzed by generalized additive models with measurements of objective skinfold thickness as x-variables and total body fat percentage (BF%) as *y*-variable.

#### Combinations of non-linear and linear relationships

**Figure 3:**
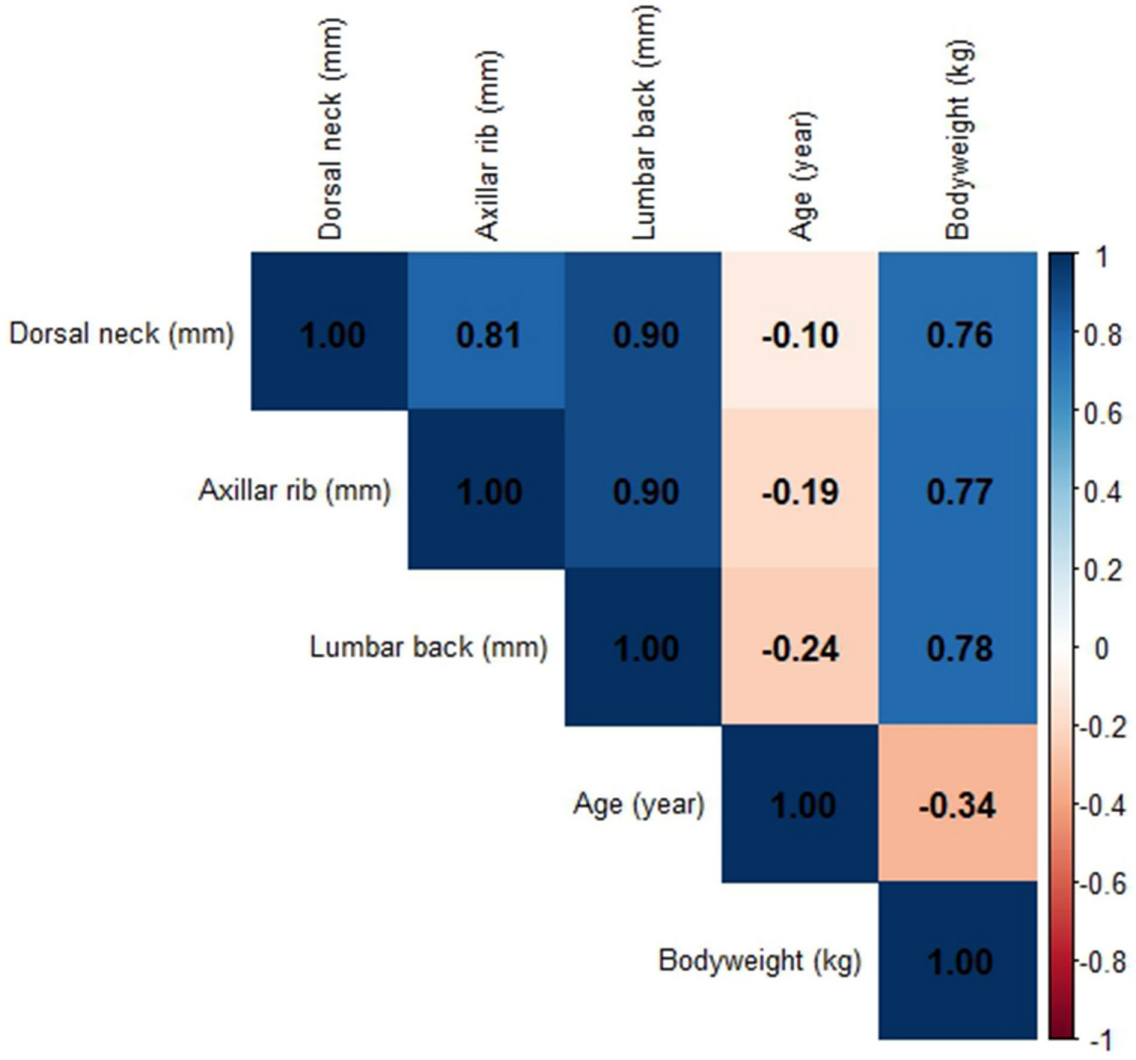
Spearman’s correlation matrix table of the numeric variables presented as a heatmap. Objective measurements of skinfold thickness (dorsal neck, axillar rib, and lumbar back) with the caliper have been entered in millimeter (mm), the variable “age” has been entered in years, and the “bodyweight” has been entered in kilograms (kg). The values within the colored squares represent the correlation coefficients, the color coding in blue represents different levels of positive correlations, and the color coding in orange represents different levels of negative correlations. The vertical bar to the right shows the level of correlation and its specific color.

A Spearman correlation matrix table of the numeric variables in the study has been included as a heatmap in Figure 3. The correlation coefficients in the heatmap showed that the “dorsal neck” and the “axillar rib” had the lowest correlation among the different locations for objective measurements of skinfold thicknesses. None of the locations for objective measurements of skinfold thicknesses were correlated to the variable “age,” and the variable “bodyweight” showed about the same correlation to all three locations for objective measurements of skinfold thickness (Figure 3).

Objective measurements of skinfold thicknesses for the separate locations were thereafter analyzed in different combinations of non-linear relationships (spline effects including interactions with bodyweight) and linear relationships (variables in linearity) as allowed by using GAM analyses. Using two locations in linearity created unwanted collinearity in the model, as did the use of two locations with added spline effects, and such combinations were therefore not applied. The three anatomical locations were therefore tested in the six different combinations possible, and the two most significant combinations are shown in Table 7. By combining non-linear and linear relationships, the explained variation of the total DEXA BF% increased. The location of the dorsal neck was best suited as a linear variable, in combination with either the axillar rib or the dorsal back in interaction with bodyweight (within a spline) (Table 7). Addition of dog intrinsic variables such as sex, age, and neutering status of the dogs did not increase the explained variation of total BF% in the most significant combined model (gam (BF% ~ s (axillar rib, by = bodyweight) + dorsal neck)). None of the dog intrinsic variables were significant in the model (*p* ≥ 0.42) and were therefore removed. The error term in the most significant model was fairly normally distributed and showed a fairly equal variance.

**Table 7:** Non-linear and linear relationships combined, of objective measurements of skinfold thickness and total body fat percentage (BF%) evaluated by dual-energy X-ray absorptiometry (DEXA) in 23 privately owned newly euthanized dogs.

Statistical model	Smooth terms (splines)		Coefficients of linear variables		Explained variation	
Splines (s), interactions (by) and linear variable in generalized additive models (GAMs)	edf splines	*p*-value splines	SE linear variables	*p*-value linear variables	*R*^2^ Adj.	DE%
gam (BF% ~ s (axillar rib, by = bodyweight) + dorsal neck)	3.33	0.0001	0.98	0.0004	0.67	73.4
gam (BF% ~ s (lumbar back, by = bodyweight) + dorsal neck)	2.92	0.0006	1.05	0.002	0.60	66.9

s, spline; by, “in interaction with”; gam, generalized additive model; edf, estimated degrees of freedom, i.e., model effect; SE, standard error; *R*^2^ Adj., coefficient of determination adjusted; BF%, total body fat percentage; DE, deviance explained, i.e., the explained variation of total BF%. Data were analyzed by generalized additive models with objective measurements of skinfold thickness as *x*-variables and total body fat percentage (BF%) as *y*-variable.

**Figure 4:**
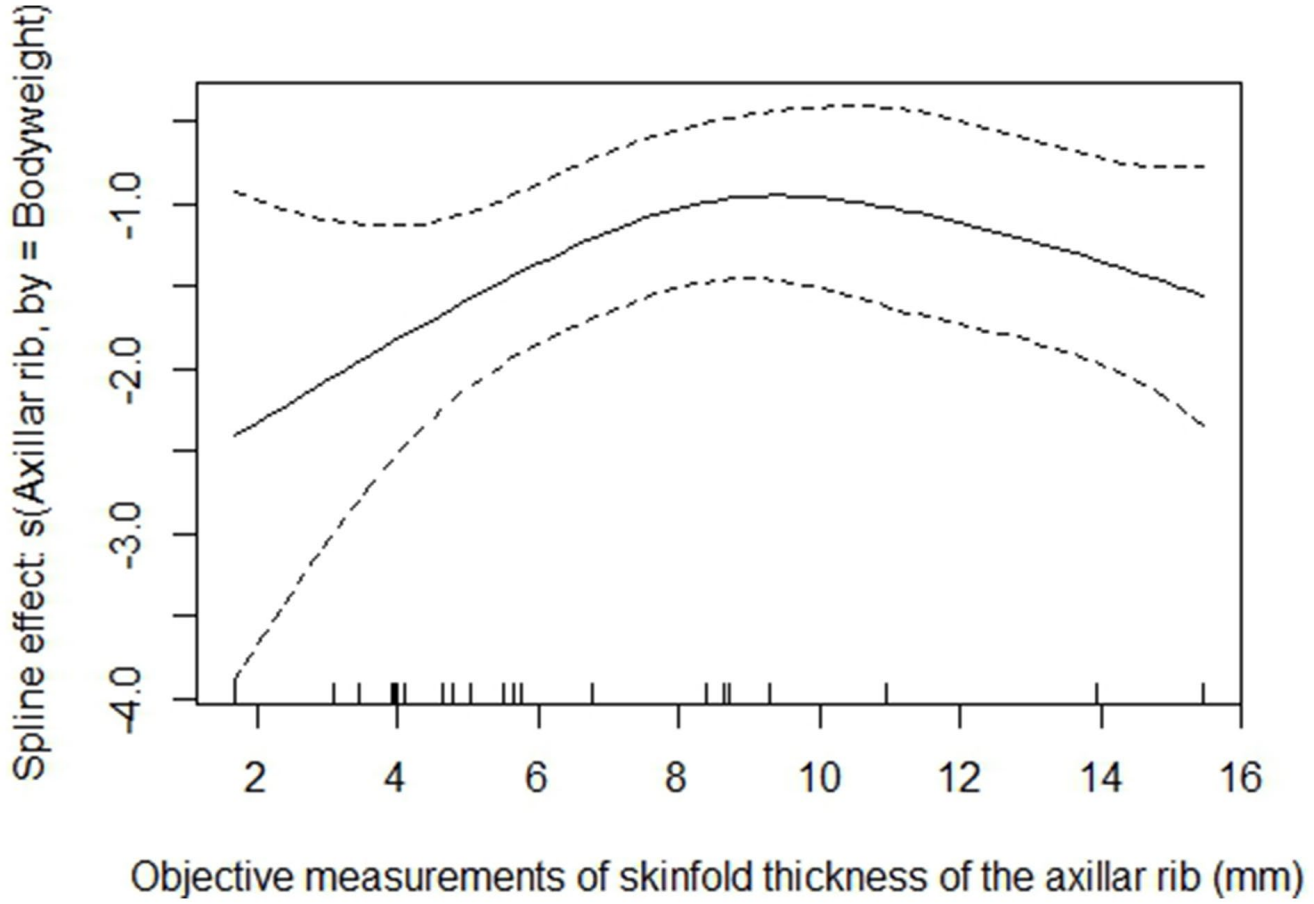
Plot of the spline effect of the objective measurements of skinfold thickness of the axillar rib in interaction with bodyweight. Objective measurements of skinfold thickness are in millimeter (mm), and the bodyweight is in kilograms (kg). On the *y*-axis, the “spline effect” (s (axillar rib, by = bodyweight)) is displayed (no unit). The range of the *y*-axis is 45% (which is the explained variation by the spline) of the variation in total body fat percentage (BF%) evaluated by dual-energy X-ray absorptiometry (DEXA). On the *x*-axis, the mean value of the measurements in triplicate from all dogs (*n* = 23) of the objective measurements of skinfold thickness at the location of the axillar rib is displayed by small vertical lines. The range of the *x*-axis is 1–16 mm. Analysis was performed with a generalized additive model (GAM), and the plot of the spline effect was generated from the most significant final model (gam (BF% ~ s (axillar rib, by = bodyweight) + dorsal neck)). The full line represents the best fit of the spline effect, and the two dotted lines represent the variation in the data. The estimated degrees of freedom (edf) of the spline effect are 3.33, indicating a cubic fit. The plot shows a significant relationship (*p* = 0.0001), starting with a linear trend and thereafter a curved shape.

The spline effect (s (axillar rib, by = bodyweight)) of the most significant combined model (Table 7) was plotted for visualization of the non-linear relationship to DEXA BF% (Figure 4). The spline effect itself accounted for slightly more than 45% of the explained variation in BF% (Table 6), and by adding the dorsal neck as a linear variable, the explained variation increased to slightly more than 73% (Table 7). As visualized in Figure 4, the interaction with bodyweight was strongest for the lower values (approximately 2 to 6 mm) of the objective measurements of skinfold thickness of the axillar rib. At cohort level, a slight variation in skinfold thickness of the lower values, found in predominantly small and/or underweight dogs, thus corresponded to a large variation in DEXA BF%.

**Figure 5:**
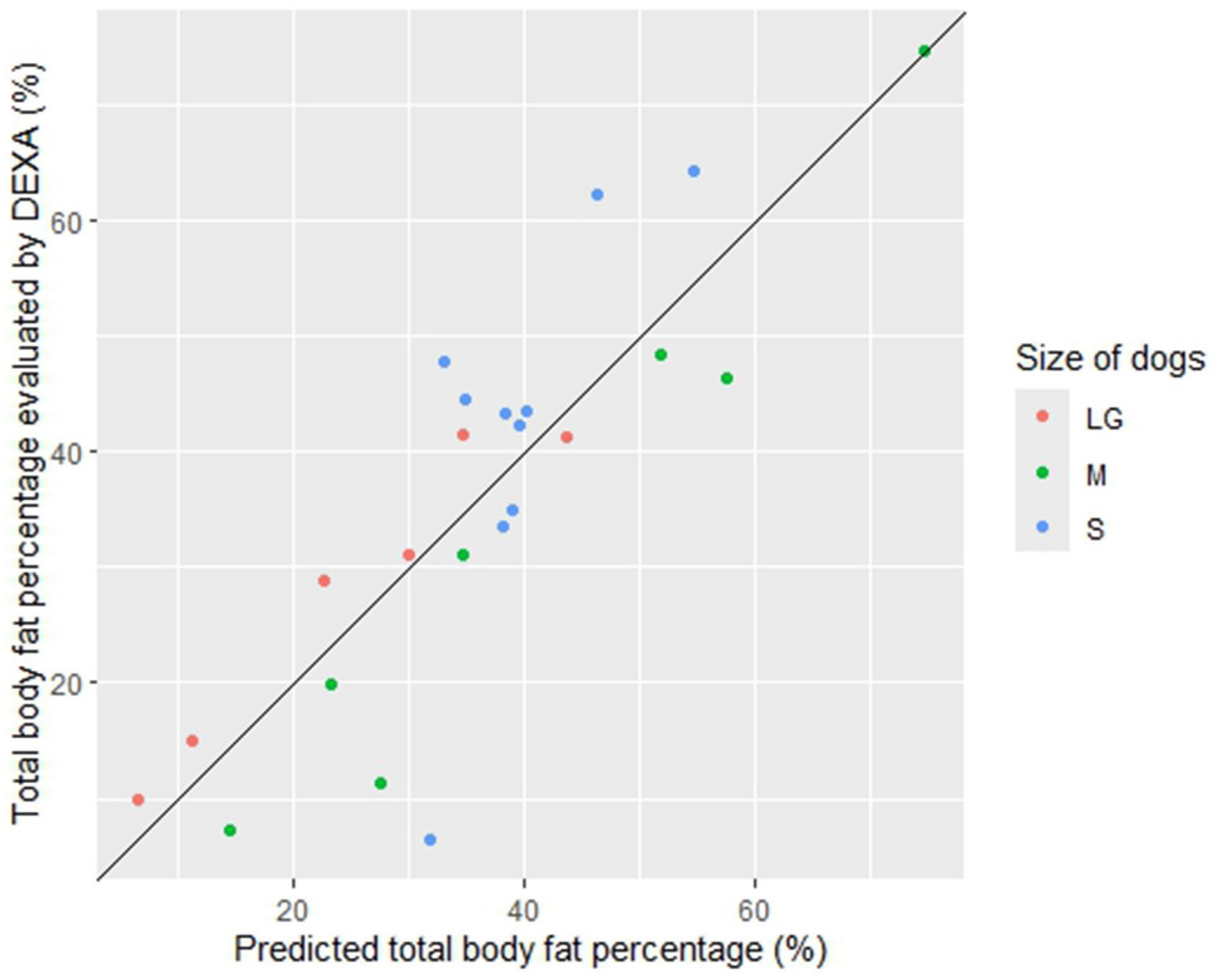
Plotted predictions of total body fat percentage (BF%) and DEXA BF%. The most significant final model (gam (BF% ~ s (axillar rib, by = bodyweight) + dorsal neck)) was used for the predictions. On the *y*-axis, the observed mean values from the replicates on BF% evaluated by dual-energy X-ray absorptiometry (DEXA) from all dogs (*n* = 23) are displayed. On the *x*-axis, the predictions of BF% from the most significant final model for each dog (*n* = 23) are displayed. The scale of both *y*- and *x*-axis is 0–80%. The dots represent individual dogs, which are color-coded according to the size of the dogs; small (S, blue) < 10 kg, medium (M, green) 10–25 kg, and large/giant (LG, red) > 25 kg. The full line represents a perfect agreement with a slope of 1. The plot indicates that BF% of dogs can be predicted with a good agreement by using the axillar rib in interaction with bodyweight (within a spline) plus the dorsal neck as a linear variable, but the BF% of small-sized dogs (blue) were over-predicted (one underweight dog) and under-predicted (three overweight to obese dogs) to a larger degree than for dogs of larger sizes.

The most significant combined model (gam (BF% ~ s (axillar rib, by = bodyweight) + dorsal neck)) (Table 7) was used as the final model to predict BF% from the objective measurements of skinfold thickness, and the predictions were plotted against the observed values of DEXA BF% (Figure 5). The plot in Figure 5 indicated that the total BF% of dogs could be predicted with a good agreement (deviance explained 73.4%) over the whole range of data by using the axillar rib in interaction with bodyweight (within a spline) plus the dorsal neck as a linear variable. Leave-one-out cross-validation (LOOCV) on the most significant combined model showed that the model’s predictions deviated from the observed values of DEXA BF% by a root mean square error (RMSE) of 11.3%. The model slightly underpredicted the BF% compared to the observed values of DEXA BF% by a mean bias of −0.24%. The 95% confidence interval (CI) of the mean prediction bias was −5.3 to +4.8%. The slope of the predicted versus the observed values on BF% was 0.92. In the prediction plot of the final model (Figure 5), the size of the dogs was color-coded, but “size” was not included in the GAM analysis. The coloring in the plot showed that the BF% of small-sized dogs were over- and under-predicted to a larger degree than for dogs of larger sizes (Figure 5).

## Discussion

In the current study, objective measurements of skinfold thickness with a caliper showed high intra- and inter-observer reliability at all anatomical locations (dorsal neck, axillar rib, and lumbar back). Subjective assessment of skinfold thicknesses with fingers differed up to 6 mm compared to objective measurements with a caliper for individual dogs, while the precision (mean SEM) of objective measurements of skinfold thickness was high for both observers. Objective measurements of skinfold thickness showed a significant non-linear relationship to DEXA BF%, and the measurements explained more than two-thirds of the variation in DEXA BF%. The location of the axillar rib in interaction with bodyweight (within a spline), plus the location of the dorsal neck as a linear variable, was the most significant final model used for prediction of BF%. The interaction with bodyweight for the objective measurements of skinfold thickness at the axillar rib was strongest for the lower values of skinfold thickness, and a slight variation of the skinfold thickness of predominantly small and/or underweight dogs therefore corresponded to a large variation in DEXA BF% at cohort level. Due to anesthesia risks to healthy animals, it was not deemed ethically acceptable to include a cohort of live dogs in the current study design. Using carcasses from newly euthanized dogs as an alternative enabled that anesthesia could be omitted in the research protocol, as DEXA scanning of dogs otherwise requires this procedure. Avoiding anesthesia minimizes potential safety hazards not only for dogs but also for the research personnel, as no exposure to anesthetic agents was present, neither radiation, and it was possible for the staff to leave the room during the scanning procedure.

### Intra- and inter-observer reliability and precision of objective measurements of skinfold thickness and comparison with subjective measurements

“The Harpenden skinfold caliper,” used for the first time on dogs in the current study, has been used for objective measurements of skinfold thickness in people for decades (45). Previous reliability studies of objective measurements of skinfold thickness with a caliper in dogs are to our knowledge lacking, but our results were comparable to or even higher than the recorded reliability of measurements of skinfold thickness in adolescents (21, 22, 24). The ICC values for all anatomical locations, both within and between observers, showed high reliability (intra-observer reliability, ≥0.991 and inter-observer reliability, ≥0.937) according to previously defined ranges (43, 46). According to the inter-observer reliability shown, a different observer may perform a re-evaluation of a dog, but according to Hume et al. (25), the same observer should always perform all caliper evaluations on a single subject, if possible. A test is considered reliable when it has small changes in the mean, a low standard error of measurement, and a high correlation between repeated evaluations (47). These properties held true for the objective measurements of skinfold thickness in the current study as no location differed in median values between observers, the standard error of measurements (mean SEM) was low (<0.2 mm), and the correlations of measurements (ICC) both within and between observers were high. The recorded precision of the caliper device on dogs was comparable to or even higher (i.e., a lower mean SEM) than the precision of measurements of skinfold thickness previously shown in adolescents (21, 22).

The measurement locations were not marked nor shaved, to mimic the circumstances of a clinical situation (48). However, it shall be remembered that the dogs were measured after euthanasia, and their position could therefore be comparable to sedated or anesthetized dogs. If objective measurements of skinfold thickness with a caliper are to be performed at a clinic as part of a nutritional assessment, they would presumably be performed in standing, awake dogs. Natural movements of awake dogs could possibly make equal placement of the caliper more challenging during measurements in triplicate or during repeated evaluations over time, even if a tape measure and palpation is used to ensure the intended position. The lifted skinfold of the euthanized dogs was kept in one hand during measurements of the objected skinfold thickness in triplicate, even though the skinfold was temporarily released when the value was recorded from the display and thereafter re-held. This procedure was used to standardize the position and height of the lifted skinfolds. In awake dogs, the skinfolds might need to be picked up and completely released for every single measurement in replicate, which could create variation in both position and height of the skinfolds. Slight variations in positioning (25, 26) and height (26) of skinfolds are factors that previously have been shown to affect measurement values obtained by a Harpenden skinfold caliper in people (26). Precision could not be calculated for the subjective assessment of skinfold thickness using only finger palpation, as the procedure could not be blinded, and therefore, the measure was recorded only once per observer. At each location, the median subjective and median objective measurements did not differ at cohort level. However, as small differences in objective measurements of skinfold thickness of lower values were associated with large differences of DEXA BF%, subjective assessment is not recommended, especially as individual dogs differed up to 6 mm when subjective and objective measurements were compared. In summary, the caliper device showed excellent reliability and precision, but objective measurements of skinfold thickness with a caliper need to be evaluated for reliability in awake, standing dogs to be able to fully conclude on the reliability for the Harpenden skinfold caliper in a clinical veterinary setting.

### Objective measurements of skinfold thickness with a caliper in dogs

Currently, there are no previous studies of objective measurements of skinfold thickness at the location of the axillar rib or the dorsal neck performed with a caliper in dogs, rendering it impossible to compare the results to other studies. To our knowledge, the study by Buzo et al. (39) is the only study available that has used a caliper (other than the “The Harpenden skinfold caliper”), for investigation of skinfold thickness in dogs, where they used the location of the lumbar back. The study included 100 dogs of 21 different breeds with a BCS of 2–9 and showed significant linear relationships of measurements of skinfold thickness to BCS and to radiographic measurements of subcutaneous fat tissue (39). In the current study, we used a different caliper device, and the measurements were compared against DEXA as the reference method, factors which could explain why the relationship of skinfold thickness at the location of the lumbar back was not linear to BF% in our data. It would have been interesting to compare the recorded median skinfold thickness at the lumbar back of the current study, with the skinfold thickness recorded by Buzo et al. (39), as the two cohorts displayed the same range in BCS and had several different breeds represented, but these data were not shown. The dorsal neck was the only location in the current study that showed a trend for linearity to the gold-standard DEXA BF%, a relationship that became significantly linear after it was combined with another location within the final model. Another possible explanation for the non-linear relationship to BF% found in the current study is that different dog breeds may display different skin thicknesses, independent of BCS. According to Zanna et al. (49) that investigated 20 intact dogs of two different breeds with ultrasound, Shar Pei dogs had twice as thick skin as Beagle dogs. Another study investigated the skin of 27 newly euthanized dogs of five different breeds (Poodles, Golden Retrievers, Shih Tzus, Pugs, and Labrador Retrievers) by histologic morphology and concluded significantly different skin thicknesses between breeds (50). Whether it is sufficient to correct only for bodyweight of dogs and not for breed in relation to objective measurements of skinfold thickness, as performed in the prediction equation suggested by our results, needs to be further investigated. Large cohorts containing a small number of dog breeds would be needed, enabling both bodyweight and breed type to be included as independent variables in the equations.

The mean height of the lifted skinfolds was numerically greater in the dorsal neck compared to the other locations, which was expected, as many dogs have loose skin in this area. The observers subjectively rated the axillar rib as the most challenging measurement location, as there was a risk of accidentally collecting the underlying muscle layer of latissimus dorsi, when performing the measurements with the caliper. One dog differed substantially between observers regarding this location, and presumably one of the observers had included the underlaying muscle layer. In a clinical situation, we believe that measurements of the lumbar back may be the most challenging, and especially in dog breeds with tighter skin, as it would be natural for dogs to sit down when skin in the lumbar area is manipulated. According to our limited clinical experience in evaluating objective skinfold thickness with a caliper in awake dogs, measuring dogs in sitting position is more challenging than in standing, as the skin of the lumbar back becomes even tighter, and the palpation of the wings of ilium also becomes more difficult. The anatomical locations for objective measurements of skinfold thickness in the current study were selected as all three represent locations where thickness of the underlying fat layer or occurrence of fat deposits is clinically evaluated in a BCS assessment of dogs (8). Previous studies have investigated the same anatomical location in dogs but with different methods. Linder et al. (51) showed that the fat layer over the fourth rib measured on radiographs was associated with BCS, and Mugnier et al. (52) showed that subcutaneous fat thickness measured with ultrasonography was prominent in the lumbar region on Labrador retriever dogs. As the BCS system is a well-established clinical assessment method for indirect evaluation of BF% in dogs (8–10, 13), it is not surprising that the objective measurements of skinfold thickness evaluated at the same anatomical locations as palpated in the BCS system showed significant relationships to DEXA BF%. A study of fit of healthy men and women showed that the inclusion of the thigh and calf skinfolds significantly enhanced the prediction of BF% (37). As obese dogs may display fat deposits on the limbs (8), it could be recommended to additionally include a measurement location on a limb, e.g., the thigh, in future studies of skinfold thickness in dogs.

### Relationship of objective measurements of skinfold thickness to total body fat percentage

Objective measurements of skinfold thickness showed a significant non-linear relationship to BF%, and the deviance explained was 73.4% of the DEXA BF%. Performance metrics generated from leave-one-out cross-validation (LOOCV) on the final model showed that the magnitude of the RMSE (11.3%) was 16% of the total range (6.4–74.7%) of the DEXA outcome. The RMSE might therefore be considered acceptable given the outcome range and sample size of the cohort and indicated a moderate precision in the model’s predictions. As the mean bias (−0.24) was close to zero, it suggested that the final model had minimal systematic bias and that the bias was not statistically significant as the CI included zero. The slope (0.92) of the predicted versus the observed values of BF% in the final model indicated that the predictions were reasonably well calibrated but slightly compressed (i.e., they may not capture the full range of the actual variation in BF%).

To our knowledge, prediction equations for BF% in dogs have been tested only for morphometric measurements other than measurements of skinfold thickness using multiple regression analyses, and the results showed significant but weak linear relationships with wide limits of agreement (13). The current study used GAM analyses for exploration of relationships. The deviance explained (DE) in a GAM analysis is comparable to the coefficient of determination (*R*^2^) and provides information about the goodness of fit in a model, i.e., the proportion of the total variation explained (53). In a Danish longitudinal cohort of 1,200 children, a strong correlation between measurements of skinfold thickness at four locations and DEXA BF% (*r* = 0.86, calculated *R*^2^ = 0.74) was shown, and the measurements of skinfold thickness outperformed BMI and waist circumference measurements in identifying children with excess fat (28). The explained variation in the Danish study was thereby equivalent to what was shown in the current study. Another study of 230 Chinese children used a prediction equation of BF% that included measurements of the triceps’ skinfold thickness and age and showed an explained variation (*R*^2^ = 0.63–0.81) that was also comparable to the current study (54). In the current final dog model, age, sex, and neutering status of dogs were not significant, nor did the addition of these three dog intrinsic factors increase the explained variation. It is possible that these variables could have been significant if the cohort had been larger and/or had included a larger proportion of neutered dogs in comparison to intact. Higher BF% have been previously documented with increasing age and in the neutered state with possible interactions with the sex of the dogs (3, 55). That prediction equations are dependent on the sex of the subject is well documented in human medicine (38, 56, 57). Age, sex, and neutering status are therefore recommended to be further evaluated as potential independent variables in future refinements of prediction equations of BF% based on objective measurements of skinfold thickness in dogs.

The addition of one measurement of skinfold thickness to another has been shown to significantly increase the explained variation in the prediction of BF% in children (29). Similarly, we combined objective measurements of two skinfolds and showed a significantly increased DE in the prediction of BF%, from a non-significant relationship to a strong relationship. In the predictions, there were indications of less accuracy in small dogs (<10 kg), in both underweight and obese states. The BF% of small dogs were over-predicted (one underweight dog) and under-predicted (three overweight to obese dogs) to a larger degree than for dogs of larger sizes. It shall be noted that the actual bodyweight was used as the interaction variable in the final model and that the size groups of the dogs were color-coded only for interpretation. Prediction equations, as compared to observed values of DEXA BF%, seem to underestimate BF% in obese people and overestimate BF% in leaner people (58), which is in accordance with the results of the small dogs. The result of the small dogs is also somehow comparable to the study by Gomes et al. (59) that used the sum of measurements from skinfold thicknesses at seven locations to predict BF% in adult athletes. The equations by Gomes et al. (59) only worked well in the middle span of BF%, and the predictions within the lowest and highest BF% quartiles showed less accuracy. It is evident that prediction equations for BF% in people have been developed for specific target groups defined by, e.g., age or fitness status, such as children (28, 54, 60), adolescents (22, 32), adults (37), or athletes (18, 59), and even so, these human studies show problems in the predictions. The current study had an inclusion criterion of ≥1 year of age, an age where most dogs are fully grown. Dogs, in contrast to people, may have fundamentally diverse body conformation between breeds. Despite the inclusion of only fully grown dogs in our cohort, bodyweight ranged from 2 to 52 kilograms. Notwithstanding the heterogeneity of the current dog cohort compared to cohorts in human studies, we found a significant relationship between objective measurements of skinfold thicknesses and DEXA BF%, correcting for bodyweight only.

To our knowledge, GAM analyses have not been frequently used in prediction equations of BF% based on measurements of skinfold thickness in people. GAM analyses, compared to multiple linear regression analyses, have the advantage of enabling the addition of “spline effects” (smooths terms) to the explanatory variables (53). A spline effect allows the relationship to take any form, and the equations are fitted by “maximum likelihood” and not by the “least square method.” In addition, GAM analyses can accommodate the interaction of two or more predictors, comparable to interactions applied in linear regression models (53). Lee (61) used a spline effect to investigate the relationship of BMI (in interaction with waist circumference) and BF% evaluated by bioelectrical impedance. As in our data, the model that contained a spline effect outperformed the linear regression models. The axillar rib in interaction with bodyweight (within a spline) showed a relationship to BF% that indicated a cubic fit (edf = 3.33). However, it is important to only interpret data within the range of observations. No dog exceeded 16 mm in skinfold thickness at this measurement location, which might be the case if an obese dog of a giant breed would have been included. Therefore, it is recommended to perform further analyses in other dog cohorts continuing using GAM models rather than cubic relationships, as the complexity of the relationship (edf) may change with other subjects included, which will be captured by the features in the GAM model.

In a study by Martín-Miguel et al. (60), GAM analyses were utilized to select significant predictors for BF% in 577 school children using basic anthropometric measures. As performed by Martín-Miguel et al. (60), it would have been beneficial to have a larger data set enabling a split of the data into two subsets, one for development of the equation and the remaining subset for validation of the equation. However, our final model performed well in cross-validation analysis, and despite the small data set for development of the equation in the current study, significant predictors for BF% were found. As shown in the plot of the spline effect (Figure 4), a slight variation in the small values of the objective measurements of skinfold thickness of the axillar rib, of predominantly underweight and/or small dogs, corresponded to a large variation in DEXA BF% at cohort level due to the interaction with bodyweight. These results thus indicate that dogs of different sizes may display different subcutaneous fat thickness over the ribs despite scoring the same BCS. This information has not previously been described to our knowledge, although it is coherent with our clinical experience of BCS assessment. However, Jeusette et al. (13) showed that the total BF% on each BCS score varied significantly between breeds, which is pointing in the same direction as our results. Taken together, this indicates that breed type and/or the bodyweight of dogs needs to be accounted for to be able to clinically assess BCS and to clinically predict BF% from objective measurements of skinfold thicknesses in dogs, with good accuracy.

### Study limitations

In this study, we investigated the relationship of objective measurements of skinfold thickness performed with a caliper and gold-standard evaluation of DEXA BF% in a cross-sectional study design and showed a non-linear relationship dependent on bodyweight. The proposed prediction equation needs to be validated in another freestanding cohort of dogs of varying sizes and BF% and of different breeds. In addition, the reliability of the objective measurements of skinfold thickness should be tested in a clinical situation, as using the device clinically would require investment in the caliper device and additional data handling using the proper equation, which may pose a challenge in the real life. The total number of dogs included in the current cohort was low, compared to the human studies presenting equivalent results of explained variations of BF% (28, 54). Anyhow, the GAM analyses in the current study found significant predictors. The extrapolation of results from human studies on the magnitude of explained variations of BF% was needed for comparison of results in this study, as to our knowledge, no equivalent canine studies are available. Interspecies differences may be present that could constitute limitations, but as the measurement locations were selected according to where dogs have been documented to accumulate fat (8, 51, 52), and as gold-standard measurements of DEXA BF% (17, 19) were performed, the study should still possess high internal validity and the results should be relevant for dogs.

In this study, dogs were DEXA scanned in ventro-dorsal position, as this position previously has been shown to produce reliable results (19, 20). A description of the positioning of dogs in the DEXA scanner is not always included in the article nor is the procedure of the data extraction. The DEXA data were extracted according to the instructions for placement of ROIs in humans (40). However, the pelvic limbs were not retracted manually during the scanning, and with dogs in ventro-dorsal position, there is a limitation on how caudally the ROI including the thorax and abdomen can be positioned, with the result that there may have been excessive abdominal tissue in the pelvic triangular ROI. However, as all DEXA data were extracted according to this procedure, the procedure itself should not have created additional variation among the dogs included in the current study.

The BCS method was developed on live dogs in a standing position (8), and it is uncertain if the BCS system can be fully used on euthanized dogs. To approximate as closely as possible to the clinical situation, the dogs were lifted to imitate a standing position when the waistline was assessed. However, the natural tone occurring in the abdominal muscles of a live dog was not present postmortem, which could have interfered with the shape of the waist. However, as DEXA BF%, and not BCS, was the response variable in all analyses, the postmortem BCS assessment should not have created any bias in the interpretations of the objective measurements of skinfold thickness.

In the current cohort, the BF% of dogs of small sizes were under- and over-predicted by the objective measurements of skinfold thickness to a larger degree than for dogs of bigger sizes. The results on DEXA BF% are built on proportions, meaning that a low percentage of bone and/or lean mass generates a larger percentage of BF%, even though a particular dog might have the same fat mass in grams as another dog. The BCS system was developed for large dog breeds (8) using DEXA BF% as reference method. It is therefore possible that evaluation of BF% by DEXA in small dogs might not be as precise as in larger dogs, but whether there is a lower boundary in bodyweight for accurate DEXA scanning of dogs has to our knowledge not been described. Whether the problem with miss-predictions of BF% in small-sized dogs adheres to the caliper device, or the DEXA reference method, remains to be further investigated. The dog cohort of the current study displayed a large variation in bodyweight and type of breeds, which could be both an advantage and a disadvantage. The method of recruitment represents the heterogenicity of dogs admitted to a veterinary clinic. Consequently, the prediction equation was developed on a canine cohort resembling a regular clinical setting. On the other hand, if a more homogenic cohort of only larger dog breeds would have been recruited, the deviance explained would probably have been even higher, but the potential problem with the predictions of BF% in small dogs would then have been undetected. To further explore the relationship of objective measurements of skinfold thickness and DEXA BF%, the data from the four Chihuahua dogs in the current dog cohort were displayed separately in a table (Supplementary File 4). According to these data, it is apparent that all objective measurements of skinfold thickness showed a numerical increase in relation to both DEXA BF% and BCS when comparing the four Chihuahua dogs only, rendering it possible that it might be the DEXA reference method and not the caliper device that created the miss-predictions for small dogs in the final model. In small children, DEXA compared to other advanced multicompartment methods tended to overestimate BF% in the obese individuals and underestimate in the leaner (62, 63), which might also be the case with the small dogs of the current study. Longitudinal studies of small dog breeds with repeated evaluations with DEXA, in combination with objective measurements of skinfold thickness with a caliper, might give further information on the usefulness of the caliper device in small dogs. With such study design, it would be possible to study not only the relationship of objective measurements of skinfold thickness and BF% in small dogs but also the correlation of the change between the prediction variables and the gold-standard outcome.

### Future clinical implications

We imagine two possible clinical implications of the caliper device. One implication could be to complement the clinical BCS assessment, to in more detail record the change of BF% in a patient with objective measurements of skinfold thickness. Future research should therefore evaluate dogs of all breeds, sizes, and BCS that either increase or decrease in body fat, with objective measurements of skinfold thickness combined with BCS assessments and recordings of bodyweight. With such study design, the longitudinal relationship of objective measurements of skinfold thickness with a caliper and BCS could be studied. Predictions of BF% are another possible clinical implication but that would require access to the prediction equation. It might also be possible that objective measurements of skinfold thickness could be included in a BCS assessment, for a more precise, indirect evaluation of BF% than using BCS only, but that needs to be investigated. However, if measurement values of skinfold thickness are followed, e.g., in a weight loss intervention, there might not be a clinical need to predict the BF% of dogs to record changes in body fat, as this study has proven that objective measurements of skinfold thickness with a caliper has a significant relationship to BF%.

## Conclusion and clinical importance

Objective measurements of skinfold thickness could be assessed with high reliability with a caliper, but the method should be further evaluated for reliability in a clinical situation on live dogs. Objective measurements of skinfold thickness showed a significant non-linear relationship to DEXA BF% and the best fitted prediction equation based on the locations of the dorsal neck and the axillar rib explained more than two-thirds of the variation in DEXA BF%. Predictions of BF% from skinfold thickness of small-sized dogs should, however, be performed with caution. Longitudinal clinical studies with larger cohorts of small-, medium-, and large-sized dogs of different breeds and BCS are warranted, to evaluate the caliper device for its potential to follow changes of BF%. The clinical use of measurement values of skinfold thickness as well as the clinical use of predictions of BF% should also be studied. Objective measurements of skinfold thickness may in the future be practically implemented in nutritional assessments of dogs visiting weight loss or nutritional clinics.

## Data Availability

The original contributions presented in the study are included in the article/Supplementary material, further inquiries can be directed to the corresponding author.
