# Supplementary material for: Objective measurements of skinfold thickness with a caliper show a significant relationship to total body fat percentage in dogs
Source: Front Vet Sci. 2025 Sep 12;12:1656855. doi: 10.3389/fvets.2025.1656855 (PMC12463608; doi:10.3389/fvets.2025.1656855)
Supplement: Supplementary file 1 [file Table_1.pdf]

Supplementary file 1; DEXA scanning procedure and DEXA data extraction.

The dogs were placed in ventro-dorsal recumbency with the thoracic limbs pulled cranially and the pelvic limbs caudally. A full body scan was performed on all dogs. The full length of the dog was measured (from head or front paws to back paws) as well as the width of the dog at the widest part. These measurements were entered in the DEXA-program before scanning, along with the bodyweight of the dog. Ten centimeters were added to the length of the dog, to make sure that the body scan contained “blank” scanning segments in the beginning and in the end of the scan. In the scanning program, "small animal" and "white female" were chosen as well as the appropriate weight-interval (<2.0 kg, 2.0-20.0 kg and >20.0 kg) according to the bodyweight of the dog. The DEXA scanning replicates for each dog were obtained in duplicate, triplicate, or quadruplicate. The dogs were not repositioned between scans.

The placement of the ”Regions of Interest” (ROI) were placed as follows for each DEXA replicate: The head was excluded; the thoracic limbs were placed in separate ROIs as were each pelvic limb; the rectangular ROI for the spine was aligned with the spine; a single ROI was placed over both the thorax and the cranial abdomen; a triangular-shaped ROI was placed over the pelvis and the caudal abdomen so that the lateral sides of the triangle went through the hip joints (see Supplementary Figure 1. below for visualization of the placements of the ROIs). Once the ROIs had been placed, the DEXA software ran the body composition analysis generating data which was exported in an XML Paper Specification (XPS file) format. This file was read and converted to a CSV file using an in-house dedicated program written in Python (3.11.4). Data was thereafter imported to a spreadsheet (Microsoft Excel). The DEXA scanning replicates entailed a repeated run of the DEXA scanner, so that new data was acquired, re-placing the ROIs was performed as was re-analysis by the software.

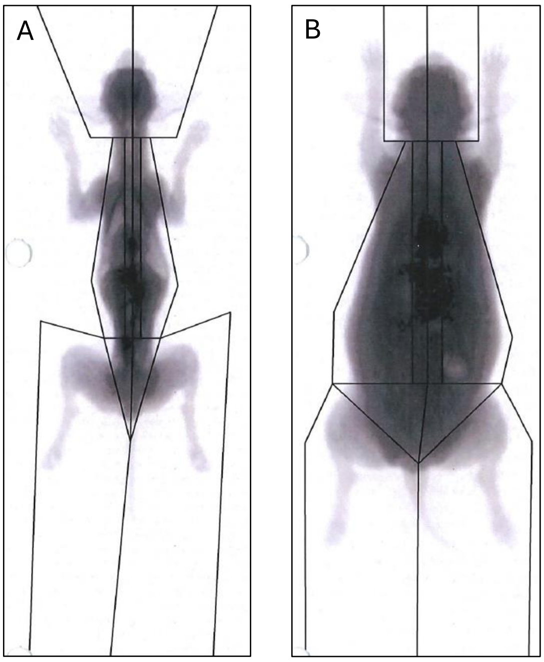

Supplementary Figure 1. Placement of ”Regions of Interest” (ROI) in two Chihuahua dogs. A) a dog with Body Condition Score (BCS) 2, and B) a dog with BCS 9 on the 9-point BCS scale (Laflamme 1997).
