# Supplementary material for: Objective measurements of skinfold thickness with a caliper show a significant relationship to total body fat percentage in dogs
Source: Front Vet Sci. 2025 Sep 12;12:1656855. doi: 10.3389/fvets.2025.1656855 (PMC12463608; doi:10.3389/fvets.2025.1656855)
Supplement: Supplementary file 3 [file Table_3.pdf]

# Supplementary file 3; R script of relationships between objective measurements of skinfold thickness and DEXA BF%

2025-07-22

```
library(tidyverse)

## ── Attaching core tidyverse packages ──────────────────────── tidyverse
2.0.0 ──
## ✔ dplyr     1.1.4     ✔ readr     2.1.5
## ✔ forcats   1.0.0     ✔ stringr   1.5.1
## ✔ ggplot2   3.5.2     ✔ tibble    3.2.1
## ✔ lubridate 1.9.4     ✔ tidyr     1.3.1
## ✔ purrr     1.0.4
## ── Conflicts ──────────────────────────────────────────
tidyverse_conflicts() ──
## ✖ dplyr::filter() masks stats::filter()
## ✖ dplyr::lag()    masks stats::lag()
## ℹ Use the conflicted package (<http://conflicted.r-lib.org/>) to force all
conflicts to become errors

dexa <- read_tsv("dexa.txt")

## Rows: 23 Columns: 24
## ── Column specification
────────────────────────────────────────────────────────
## Delimiter: "\t"
## chr  (3): sex, neutering, size
## dbl (21): ID, DEXA_fat, BCS, skinfold_neck, skinfold_axilla,
skinfold_back, ...
##
## ℹ Use `spec()` to retrieve the full column specification for this data.
## ℹ Specify the column types or set `show_col_types = FALSE` to quiet this
message.

dexa$size<-as.factor(dexa$size)
dexa$neutering<-as.factor(dexa$neutering)
dexa$sex<-as.factor(dexa$sex)

dexa %>%
ggplot(aes(x=neutering, y=DEXA_fat)) +
geom_point()
```

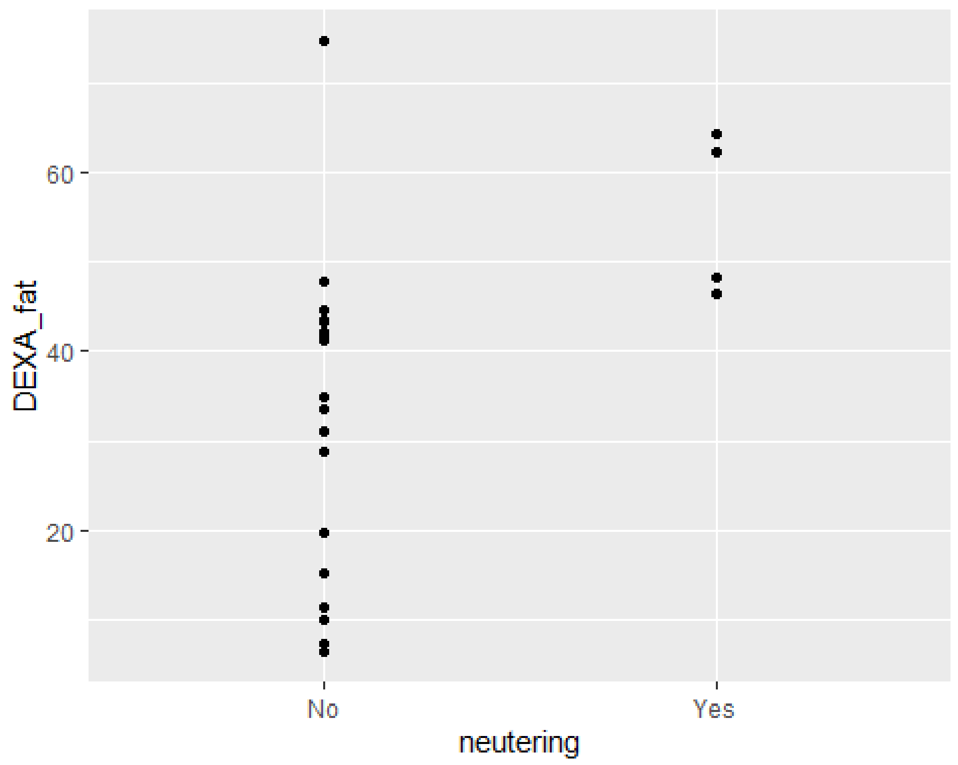


```
dexa %>%
ggplot(aes(x=sex, y=DEXA_fat)) +
geom_point()
```

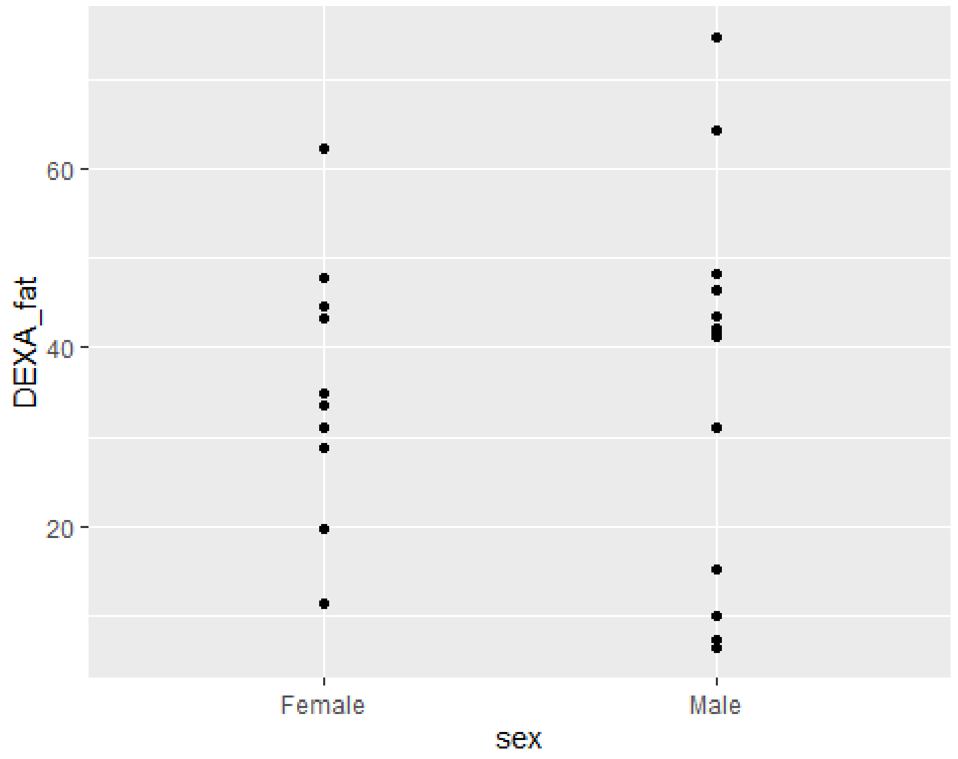

```
dexa %>%
ggplot(aes(x=age, y=DEXA_fat)) +
geom_point()
```

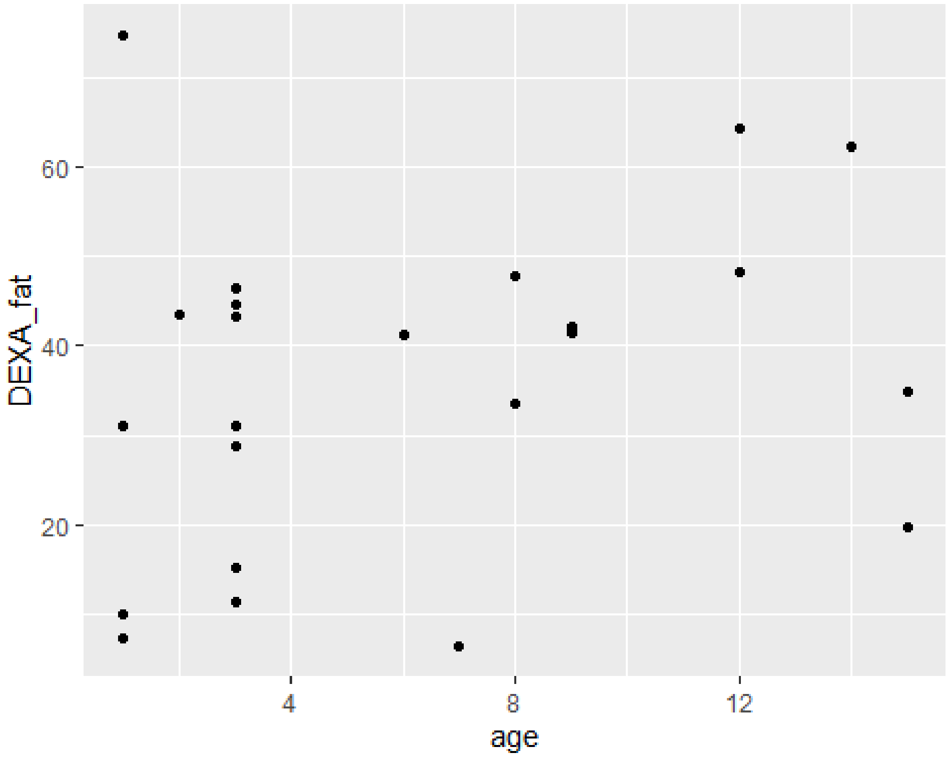


```
dexa %>%
ggplot(aes(x=skinfold_neck, y=DEXA_fat)) +
geom_point()
```

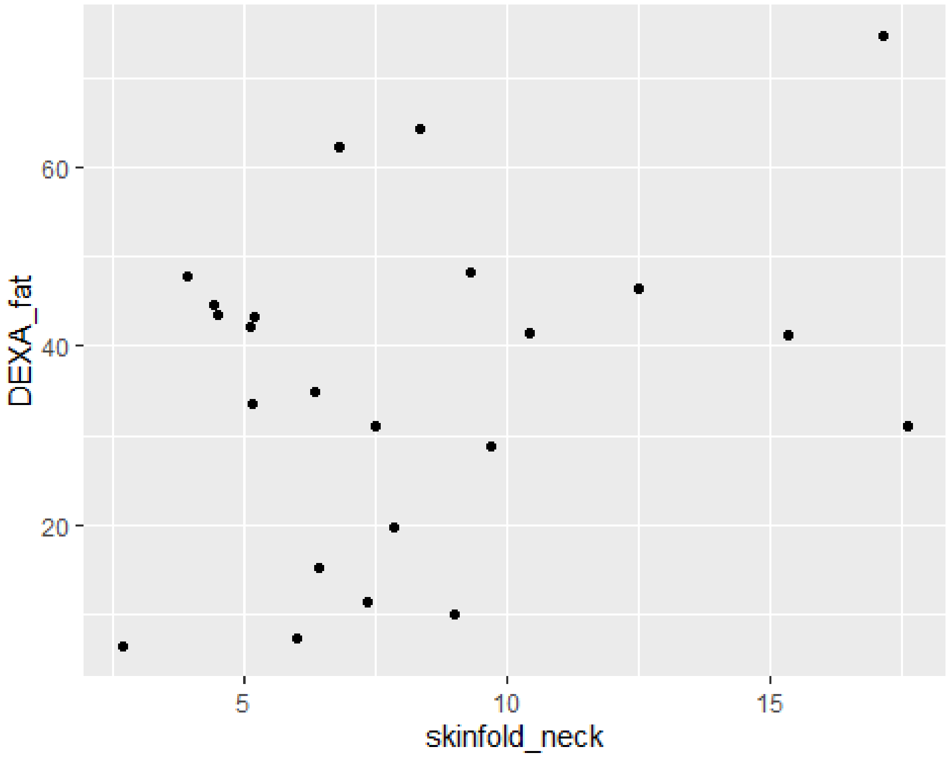


```
dexa %>%
ggplot(aes(x=skinfold_axilla, y=DEXA_fat)) +
geom_point()
```

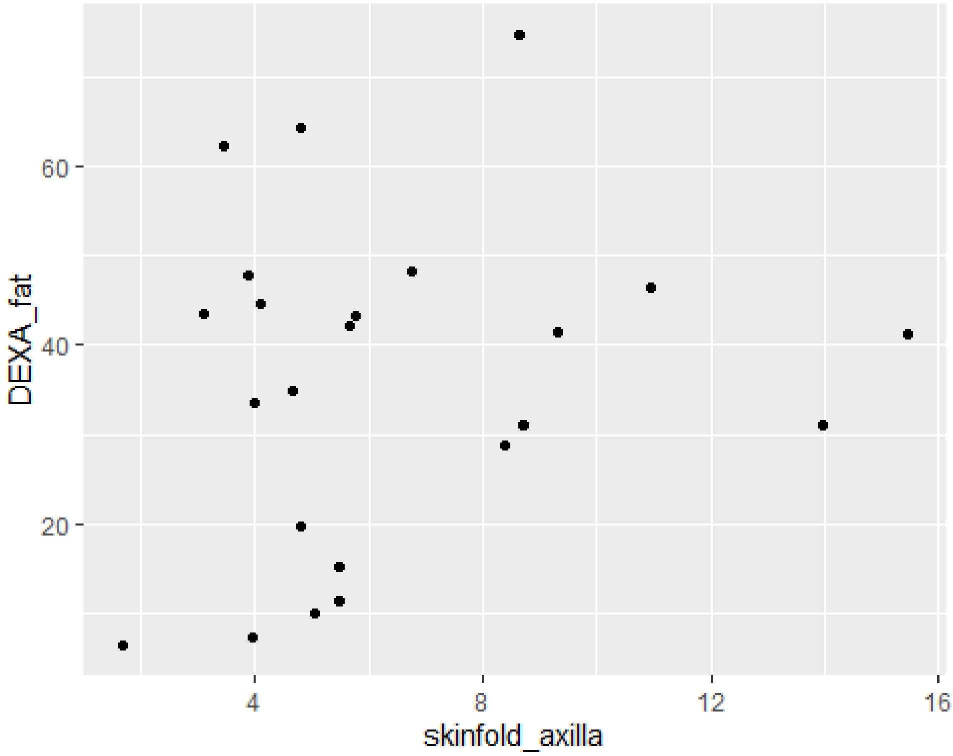

```r
dexa %>%
ggplot(aes(x=skinfold_back, y=DEXA_fat)) +
geom_point()
```

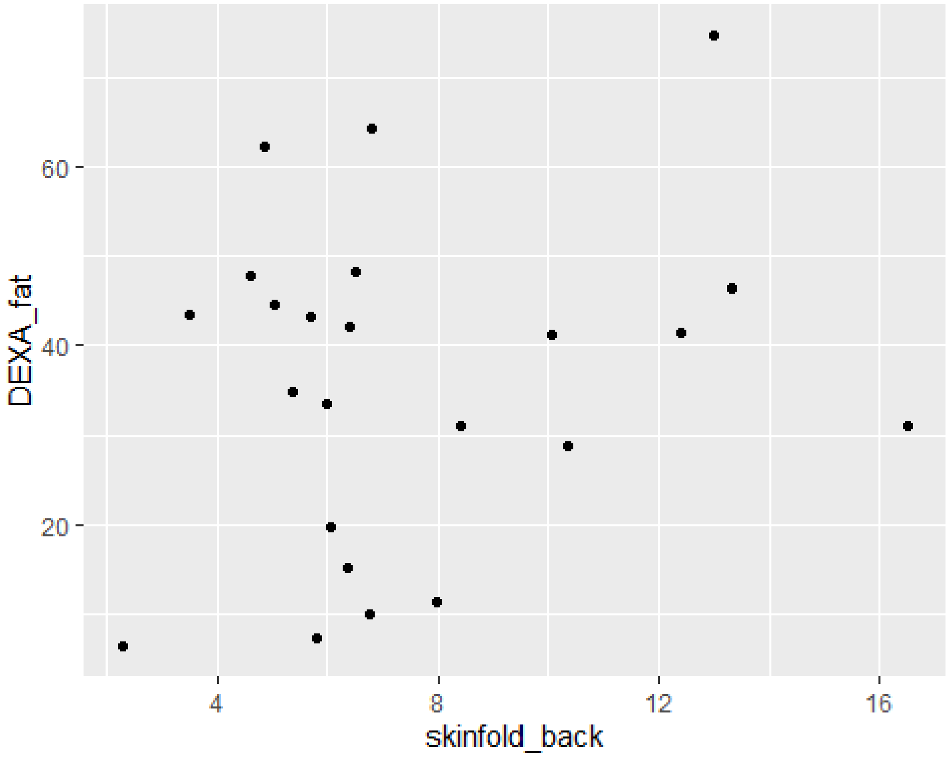


```r
dexa.lm1<-lm(DEXA_fat~skinfold_neck, data=dexa)
summary(dexa.lm1)

##
## Call:
## lm(formula = DEXA_fat ~ skinfold_neck, data = dexa)
##
## Residuals:
##     Min      1Q  Median      3Q     Max
## -27.127 -16.758   1.573  11.829  28.145
##
## Coefficients:
##               Estimate Std. Error t value Pr(>|t|)
## (Intercept)    24.9705     8.5607   2.917  0.00824 **
## skinfold_neck   1.3507     0.9388   1.439  0.16497
## ---
## Signif. codes:  0 '***' 0.001 '**' 0.01 '*' 0.05 '.' 0.1 ' ' 1
##
## Residual standard error: 17.98 on 21 degrees of freedom
## Multiple R-squared:  0.08972,    Adjusted R-squared:  0.04638
## F-statistic:  2.07 on 1 and 21 DF,  p-value: 0.165
```

```
dexa.lm2<-lm(DEXA_fat~skinfold_axilla, data=dexa)
summary(dexa.lm2)

##
## Call:
## lm(formula = DEXA_fat ~ skinfold_axilla, data = dexa)
##
## Residuals:
##    Min     1Q Median     3Q    Max
## -26.33 -13.50   0.59  10.71  36.51
##
## Coefficients:
##                 Estimate Std. Error t value Pr(>|t|)
## (Intercept)      29.7952     8.3422   3.572   0.0018 **
## skinfold_axilla   0.9708     1.1488   0.845   0.4076
## ---
## Signif. codes:  0 '***' 0.001 '**' 0.01 '*' 0.05 '.' 0.1 ' ' 1
##
## Residual standard error: 18.53 on 21 degrees of freedom
## Multiple R-squared:  0.03289,    Adjusted R-squared:  -0.01316
## F-statistic: 0.7142 on 1 and 21 DF,  p-value: 0.4076

dexa.lm3<-lm(DEXA_fat~skinfold_back, data=dexa)
summary(dexa.lm3)

##
## Call:
## lm(formula = DEXA_fat ~ skinfold_back, data = dexa)
##
## Residuals:
##     Min      1Q  Median      3Q     Max
## -26.763 -14.813   1.342  11.650  32.549
##
## Coefficients:
##               Estimate Std. Error t value Pr(>|t|)
## (Intercept)     27.548      9.322   2.955  0.00755 **
## skinfold_back    1.123      1.123   1.000  0.32865
## ---
## Signif. codes:  0 '***' 0.001 '**' 0.01 '*' 0.05 '.' 0.1 ' ' 1
##
## Residual standard error: 18.41 on 21 degrees of freedom
## Multiple R-squared:  0.04546,    Adjusted R-squared:  9.487e-06
## F-statistic:     1 on 1 and 21 DF,  p-value: 0.3286

plot(dexa.lm1)
```

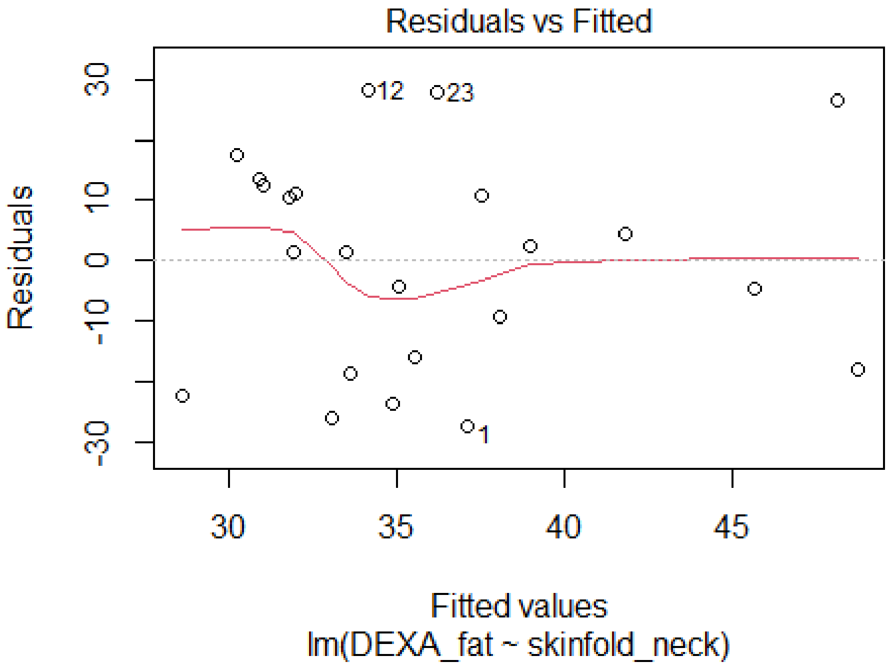

Residuals vs Fitted
Residuals
Fitted values
lm(DEXA_fat ~ skinfold_neck)


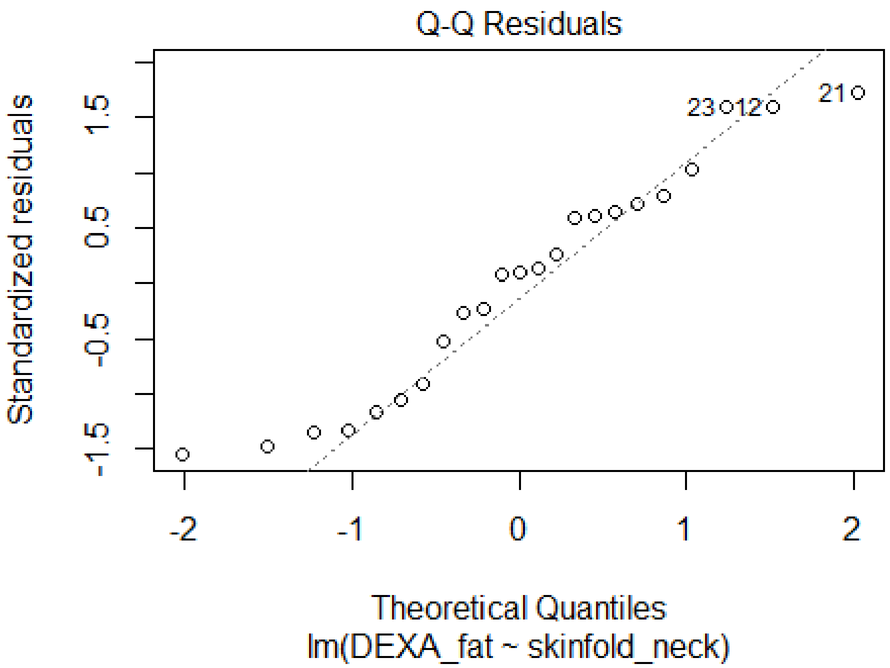

Q-Q Residuals
Standardized residuals
Theoretical Quantiles
lm(DEXA_fat ~ skinfold_neck)

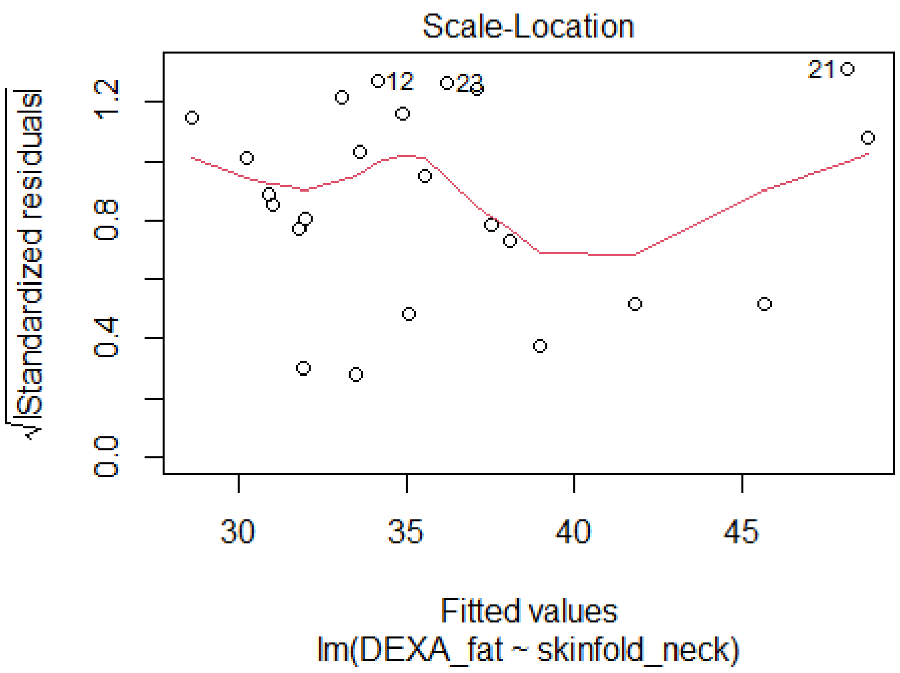


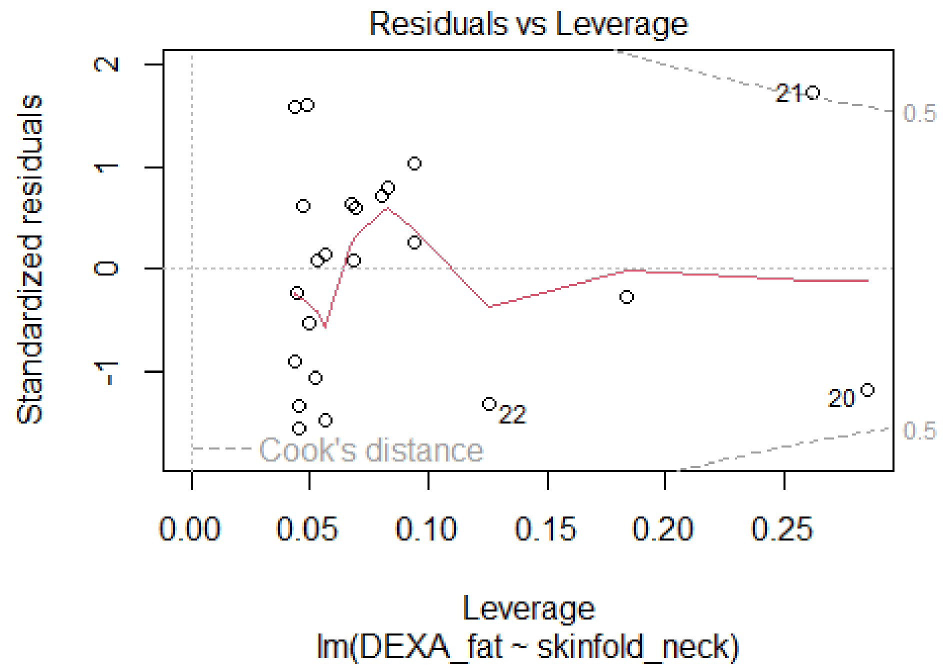


```
plot(dexa.lm2)
```

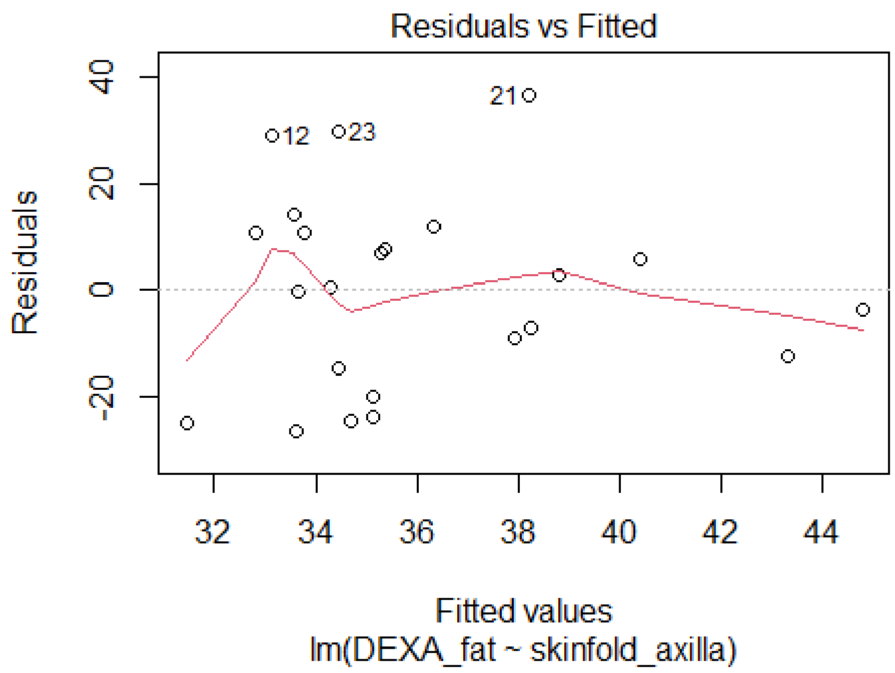
Residuals vs Fitted
Residuals
40
20
0
-20
21
12
23
32
34
36
38
40
42
44
Fitted values
lm(DEXA_fat ~ skinfold_axilla)

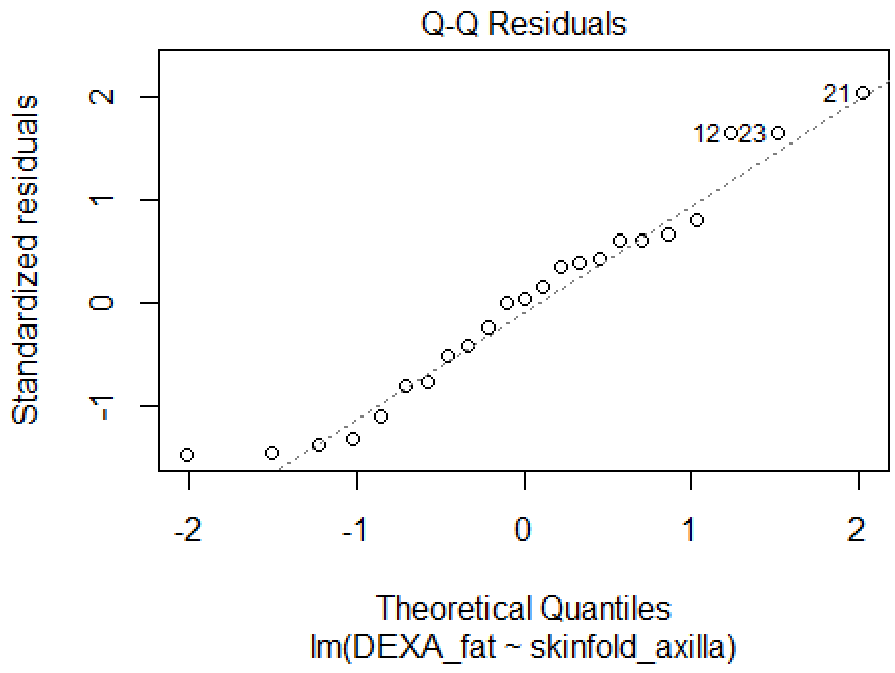
Q-Q Residuals
Standardized residuals
2
1
0
-1
21
12
23
-2
-1
0
1
2
Theoretical Quantiles
lm(DEXA_fat ~ skinfold_axilla)

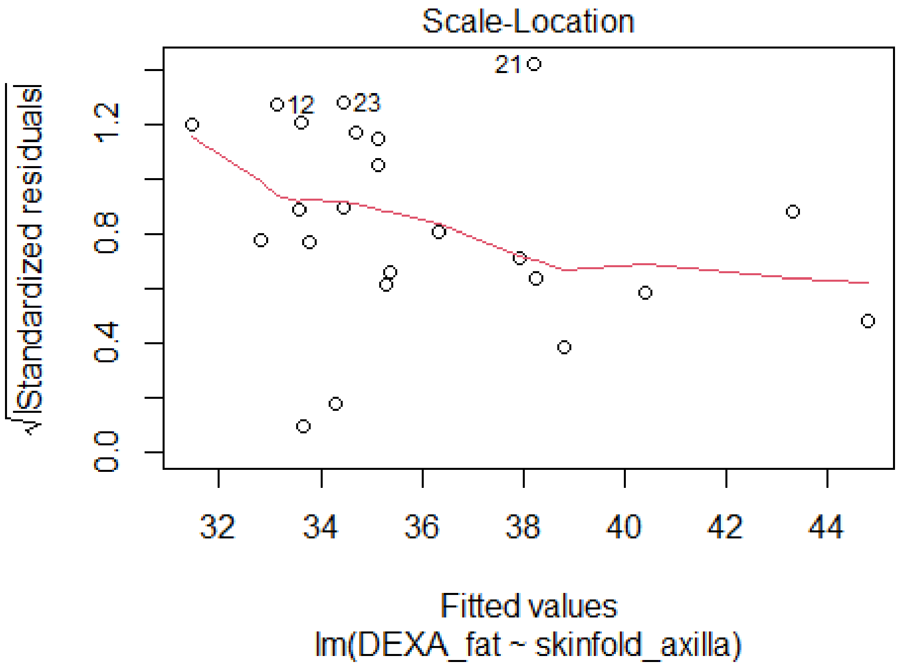


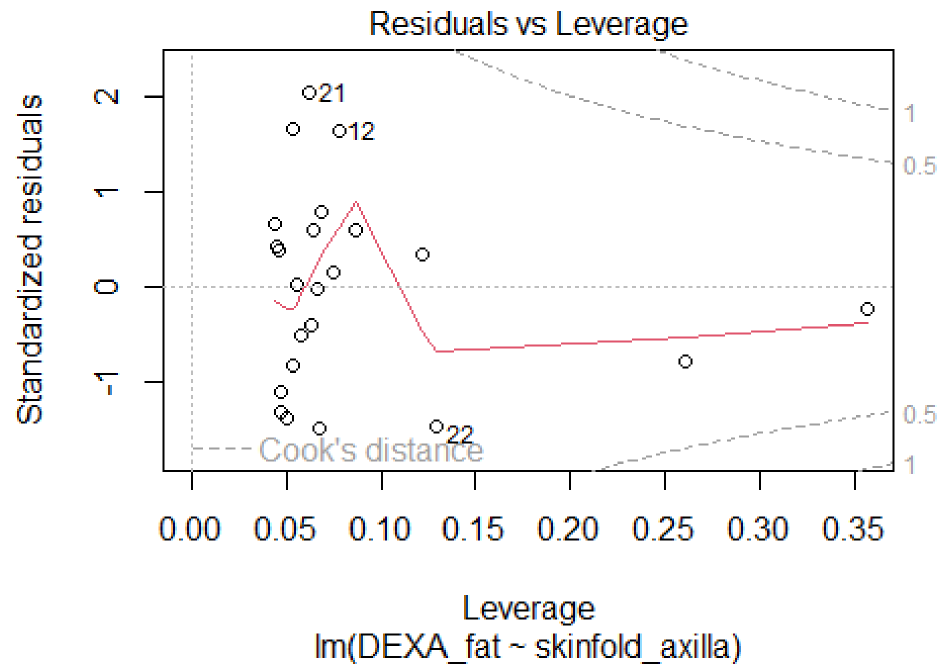


```
plot(dexa.lm3)
```

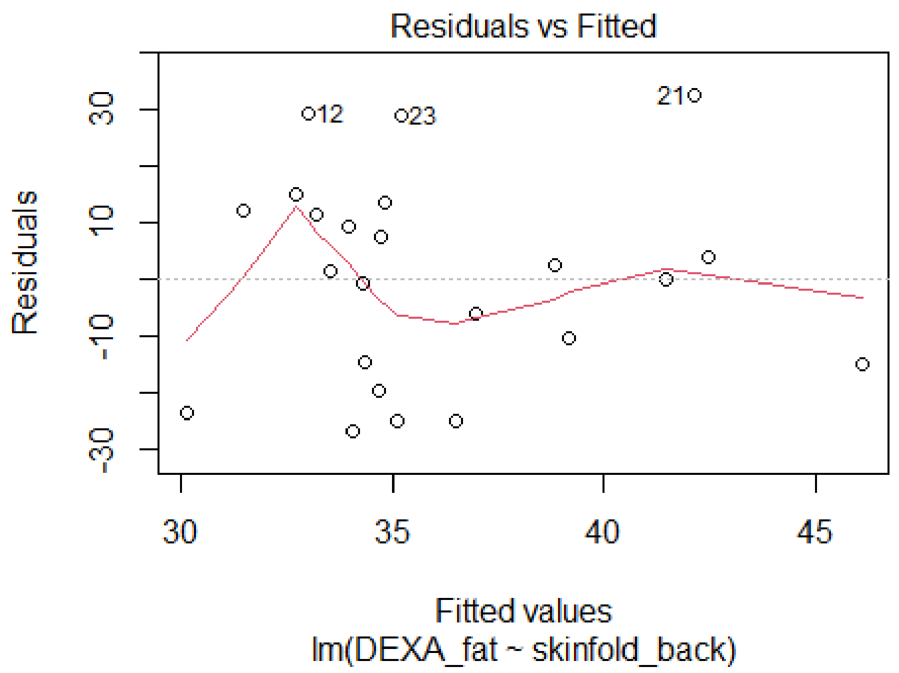
Residuals vs Fitted
Residuals
30
10
-10
-30
12
23
21
30
35
40
45
Fitted values
lm(DEXA_fat ~ skinfold_back)

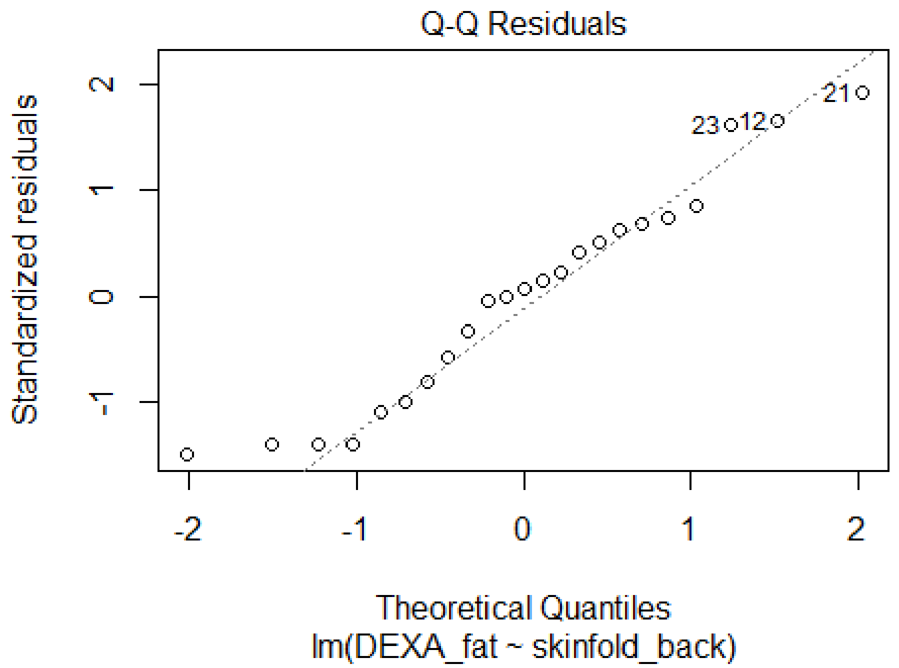
Q-Q Residuals
Standardized residuals
2
1
0
-1
23
12
21
-2
-1
0
1
2
Theoretical Quantiles
lm(DEXA_fat ~ skinfold_back)

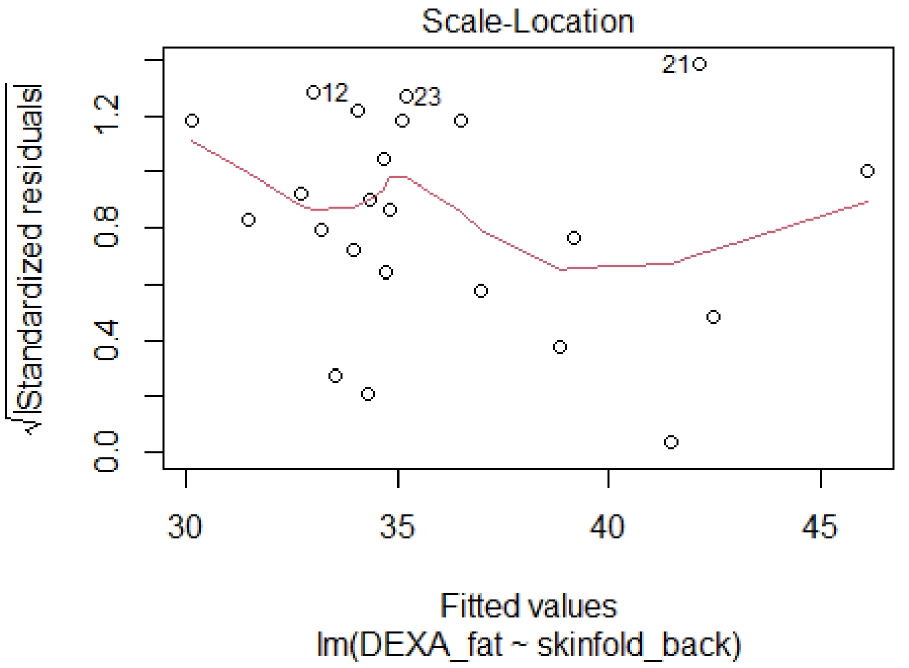


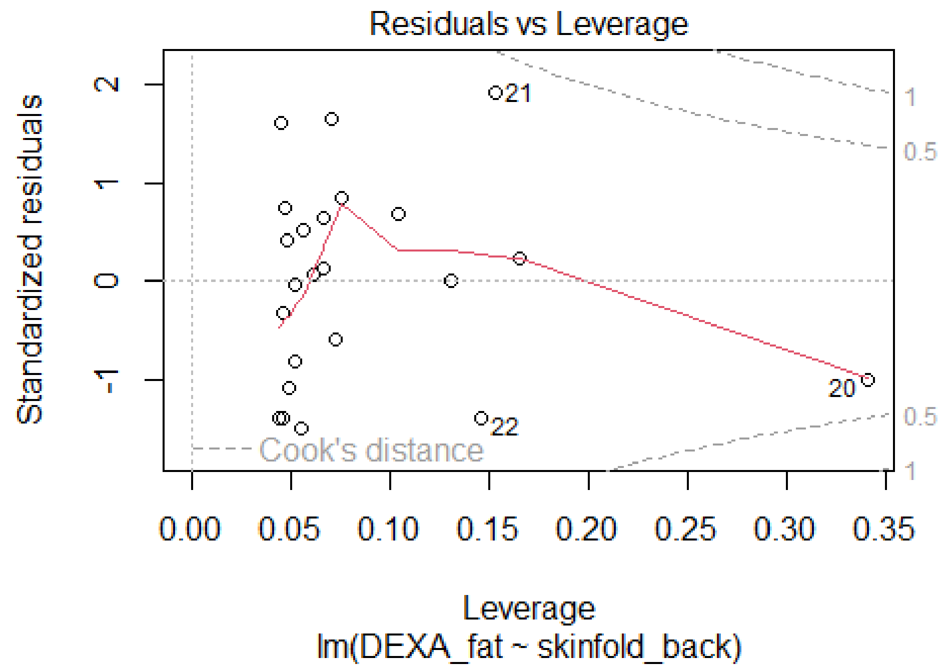


```
dexa %>%
  ggplot(aes(x = skinfold_neck, y = DEXA_fat)) +
  geom_point() +
  stat_smooth(method = "lm") +
```

```
  labs(
    x = "Objective measurements of skinfold thickness of the dorsal neck
(mm)",
    y = "Total body fat percentage evaluated by DEXA (%)"
  )

## `geom_smooth()` using formula = 'y ~ x'
```

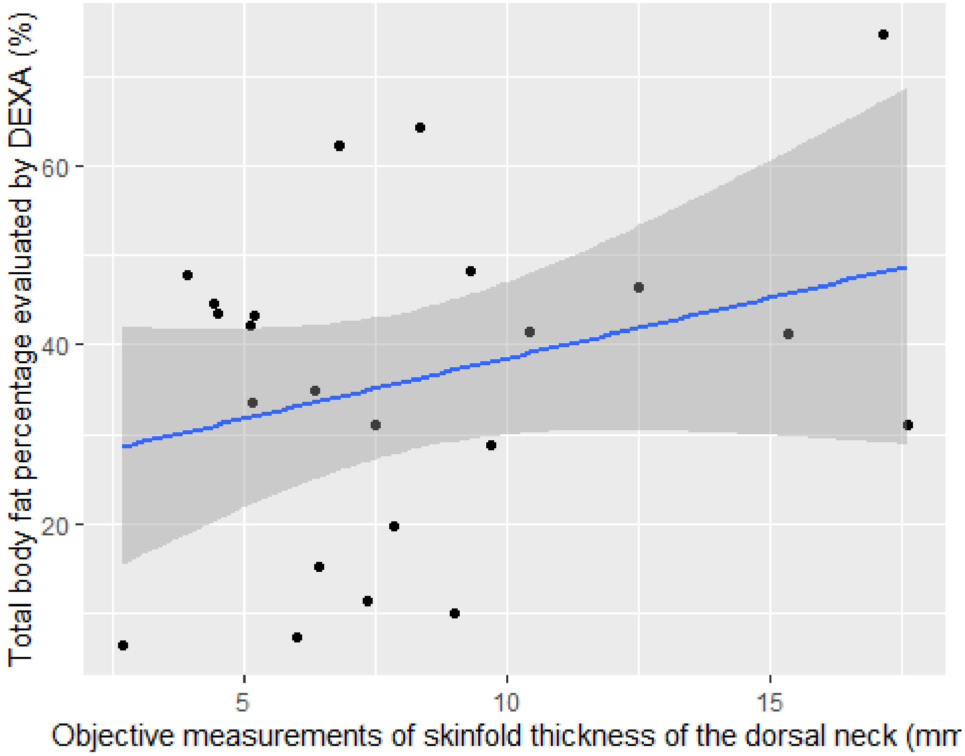


```
dexa %>%
ggplot(aes(x=skinfold_axilla, y=DEXA_fat)) +
geom_point() +
stat_smooth(method="lm")

## `geom_smooth()` using formula = 'y ~ x'
```

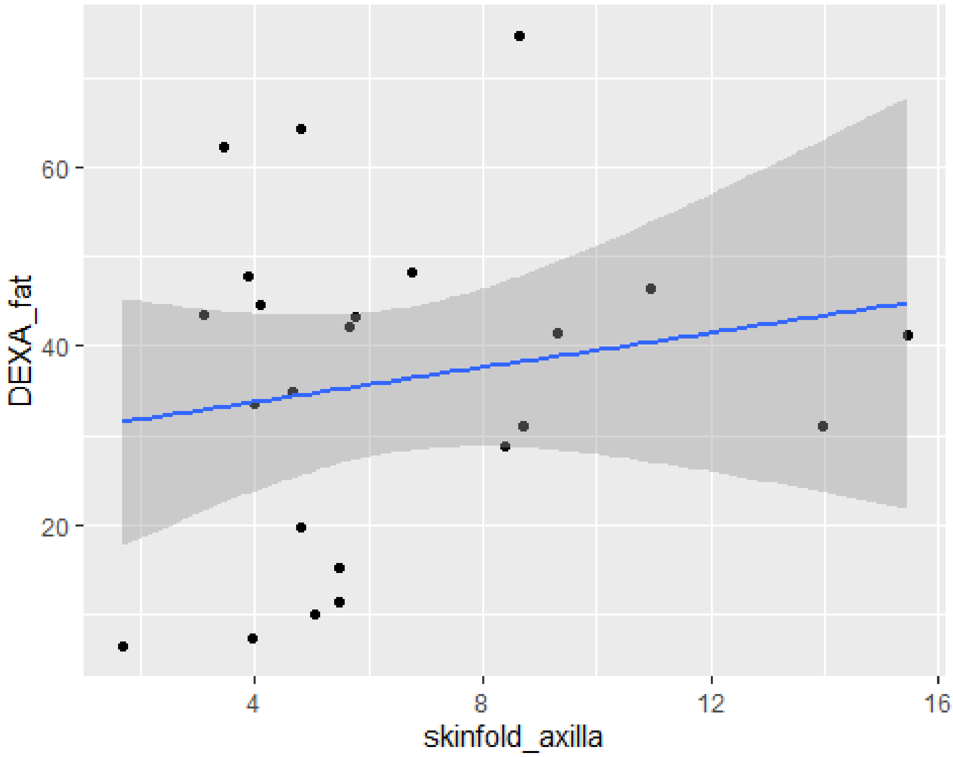


```
dexa %>%
ggplot(aes(x=skinfold_back, y=DEXA_fat)) +
geom_point() +
stat_smooth(method="lm")

## `geom_smooth()` using formula = 'y ~ x'
```

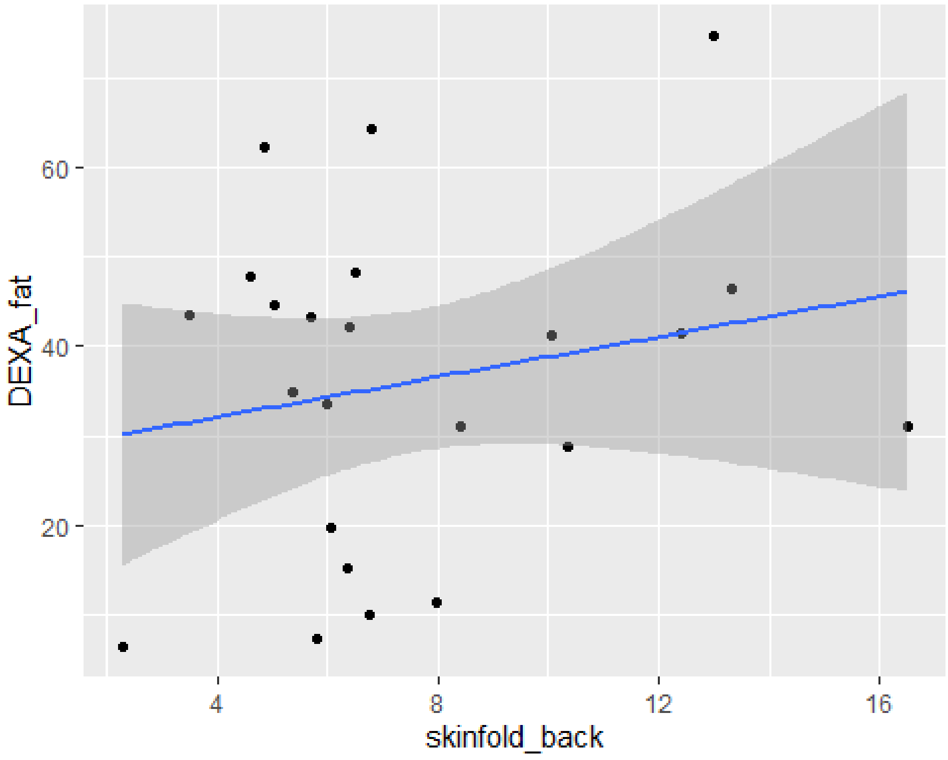


```
library(mgcv)

## Warning: package 'mgcv' was built under R version 4.4.3

## Loading required package: nlme

##
## Attaching package: 'nlme'

## The following object is masked from 'package:dplyr':
##
##     collapse

## This is mgcv 1.9-3. For overview type 'help("mgcv-package")'.

dexa.gam1<-gam(DEXA_fat~s(skinfold_neck) ,
method="REML", data=dexa)
options(scipen=999)
summary(dexa.gam1)

##
## Family: gaussian
## Link function: identity
##
## Formula:
## DEXA_fat ~ s(skinfold_neck)
##
## Parametric coefficients:
```

```
##              Estimate Std. Error t value      Pr(>|t|)
## (Intercept)   36.043      3.748   9.616 0.00000000383 ***
## ---
## Signif. codes:  0 '***' 0.001 '**' 0.01 '*' 0.05 '.' 0.1 ' ' 1
##
## Approximate significance of smooth terms:
##                  edf Ref.df    F p-value
## s(skinfold_neck)   1      1 2.07   0.165
##
## R-sq.(adj) =  0.0464   Deviance explained = 8.97%
## -REML = 93.603  Scale est. = 323.14    n = 23
```

```
dexa.gam2<-gam(DEXA_fat~s(skinfold_axilla) ,
method="REML", data=dexa)
options(scipen=999)
summary(dexa.gam2)
```

```
##
## Family: gaussian
## Link function: identity
##
## Formula:
## DEXA_fat ~ s(skinfold_axilla)
##
## Parametric coefficients:
##              Estimate Std. Error t value      Pr(>|t|)
## (Intercept)   36.043      3.864   9.329 0.00000000644 ***
## ---
## Signif. codes:  0 '***' 0.001 '**' 0.01 '*' 0.05 '.' 0.1 ' ' 1
##
## Approximate significance of smooth terms:
##                    edf Ref.df     F p-value
## s(skinfold_axilla)   1  1.001 0.714   0.408
##
## R-sq.(adj) =  -0.0132   Deviance explained = 3.29%
## -REML = 94.239  Scale est. = 343.32    n = 23
```

```
dexa.gam3<-gam(DEXA_fat~s(skinfold_back) ,
method="REML", data=dexa)
options(scipen=999)
summary(dexa.gam3)
```

```
##
## Family: gaussian
## Link function: identity
##
## Formula:
## DEXA_fat ~ s(skinfold_back)
##
## Parametric coefficients:
##              Estimate Std. Error t value      Pr(>|t|)
```

```
## (Intercept)   36.043      3.838    9.39 0.00000000576 ***
## ---
## Signif. codes:  0 '***' 0.001 '**' 0.01 '*' 0.05 '.' 0.1 ' ' 1
##
## Approximate significance of smooth terms:
##                  edf Ref.df F p-value
## s(skinfold_back)   1      1 1   0.329
##
## R-sq.(adj) =  6.48e-06   Deviance explained = 4.55%
## -REML = 94.102  Scale est. = 338.86    n = 23
```

```
dexa.gam11<-gam(DEXA_fat~s(skinfold_neck, by=weight) ,
method="REML", data=dexa)
options(scipen=999)
summary(dexa.gam11)
```

```
##
## Family: gaussian
## Link function: identity
##
## Formula:
## DEXA_fat ~ s(skinfold_neck, by = weight)
##
## Parametric coefficients:
##             Estimate Std. Error t value     Pr(>|t|)
## (Intercept)   51.395      5.584   9.204 0.0000000199 ***
## ---
## Signif. codes:  0 '***' 0.001 '**' 0.01 '*' 0.05 '.' 0.1 ' ' 1
##
## Approximate significance of smooth terms:
##                          edf Ref.df     F p-value
## s(skinfold_neck):weight 3.02  3.484 4.465  0.0138 *
## ---
## Signif. codes:  0 '***' 0.001 '**' 0.01 '*' 0.05 '.' 0.1 ' ' 1
##
## R-sq.(adj) =   0.38   Deviance explained = 46.5%
## -REML = 93.123  Scale est. = 210.16    n = 23
```

```
dexa.gam22<-gam(DEXA_fat~s(skinfold_axilla, by=weight) ,
method="REML", data=dexa)
options(scipen=999)
summary(dexa.gam22)
```

```
##
## Family: gaussian
## Link function: identity
##
## Formula:
## DEXA_fat ~ s(skinfold_axilla, by = weight)
##
## Parametric coefficients:
```

```
##              Estimate Std. Error t value     Pr(>|t|)
## (Intercept)   49.357      5.358   9.212 0.0000000209 ***
## ---
## Signif. codes:  0 '***' 0.001 '**' 0.01 '*' 0.05 '.' 0.1 ' ' 1
##
## Approximate significance of smooth terms:
##                                edf Ref.df     F p-value
## s(skinfold_axilla):weight 3.163  3.617 4.056   0.019 *
## ---
## Signif. codes:  0 '***' 0.001 '**' 0.01 '*' 0.05 '.' 0.1 ' ' 1
##
## R-sq.(adj) =  0.361   Deviance explained = 45.3%
## -REML = 93.687  Scale est. = 216.41    n = 23
```

```
dexa.gam33<-gam(DEXA_fat~s(skinfold_back, by=weight) ,
method="REML", data=dexa)
options(scipen=999)
summary(dexa.gam33)
```

```
##
## Family: gaussian
## Link function: identity
##
## Formula:
## DEXA_fat ~ s(skinfold_back, by = weight)
##
## Parametric coefficients:
##              Estimate Std. Error t value    Pr(>|t|)
## (Intercept)   50.283      5.467   9.198 0.000000021 ***
## ---
## Signif. codes:  0 '***' 0.001 '**' 0.01 '*' 0.05 '.' 0.1 ' ' 1
##
## Approximate significance of smooth terms:
##                              edf Ref.df     F p-value
## s(skinfold_back):weight 3.123  3.546 4.621  0.0145 *
## ---
## Signif. codes:  0 '***' 0.001 '**' 0.01 '*' 0.05 '.' 0.1 ' ' 1
##
## R-sq.(adj) =  0.385   Deviance explained = 47.2%
## -REML = 93.253  Scale est. = 208.33    n = 23
```

```
library(corrplot)
```

```
## Warning: package 'corrplot' was built under R version 4.4.3
```

```
## corrplot 0.95 loaded
```

```
cor_matrix <- cor(dexa[, c("skinfold_neck", "skinfold_axilla",
"skinfold_back", "age", "weight")],
                  method = "spearman")
print(cor_matrix)
```

```
##                 skinfold_neck skinfold_axilla skinfold_back        age
## skinfold_neck       1.0000000       0.8091944     0.8962451 -0.1045914
## skinfold_axilla     0.8091944       1.0000000     0.8996541 -0.1906718
## skinfold_back       0.8962451       0.8996541     1.0000000 -0.2423704
## age                -0.1045914      -0.1906718    -0.2423704  1.0000000
## weight              0.7647060       0.7725025     0.7770639 -0.3353106
##                     weight
## skinfold_neck    0.7647060
## skinfold_axilla  0.7725025
## skinfold_back    0.7770639
## age             -0.3353106
## weight           1.0000000

custom_labels <- c("Dorsal neck (mm)", "Axillar rib (mm)", "Lumbar back
(mm)", "Age (year)", "Bodyweight (kg)")
colnames(cor_matrix) <- custom_labels
rownames(cor_matrix) <- custom_labels

corrplot(cor_matrix,
         method = "color",
         type = "upper",
         tl.col = "black",
         tl.cex = 0.8,
         addCoef.col = "black")
```

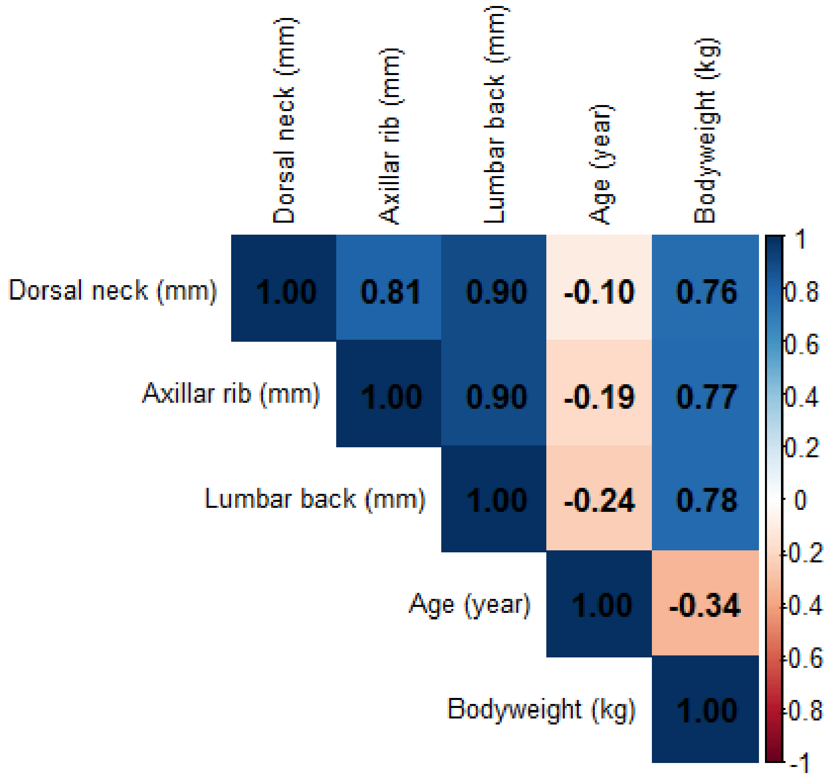


```
dexa.gam.combined1<-gam(DEXA_fat~skinfold_back + s(skinfold_neck, by=weight)
,
```

```r
method="REML", data=dexa)
options(scipen=999)
summary(dexa.gam.combined1)

## 
## Family: gaussian 
## Link function: identity 
## 
## Formula:
## DEXA_fat ~ skinfold_back + s(skinfold_neck, by = weight)
## 
## Parametric coefficients:
##               Estimate Std. Error t value Pr(>|t|)  
## (Intercept)     26.066     10.187   2.559   0.0195 *
## skinfold_back    4.579      1.679   2.727   0.0137 *
## ---
## Signif. codes:  0 '***' 0.001 '**' 0.01 '*' 0.05 '.' 0.1 ' ' 1
## 
## Approximate significance of smooth terms:
##                             edf Ref.df   F p-value   
## s(skinfold_neck):weight 2.64  3.019 7.8  0.0015 **
## ---
## Signif. codes:  0 '***' 0.001 '**' 0.01 '*' 0.05 '.' 0.1 ' ' 1
## 
## R-sq.(adj) =  0.509   Deviance explained = 59.1%
## -REML = 88.506  Scale est. = 166.27    n = 23

dexa.gam.combined2<-gam(DEXA_fat~skinfold_neck + s(skinfold_back, by=weight)
,
method="REML", data=dexa)
options(scipen=999)
summary(dexa.gam.combined2)

## 
## Family: gaussian 
## Link function: identity 
## 
## Formula:
## DEXA_fat ~ skinfold_neck + s(skinfold_back, by = weight)
## 
## Parametric coefficients:
##               Estimate Std. Error t value Pr(>|t|)   
## (Intercept)     28.538      7.541   3.785  0.00135 **
## skinfold_neck    3.680      1.046   3.520  0.00243 **
## ---
## Signif. codes:  0 '***' 0.001 '**' 0.01 '*' 0.05 '.' 0.1 ' ' 1
## 
## Approximate significance of smooth terms:
##                              edf Ref.df     F  p-value    
## s(skinfold_back):weight 2.918  3.311 8.823 0.000587 ***
## ---
```

```
## Signif. codes:  0 '***' 0.001 '**' 0.01 '*' 0.05 '.' 0.1 ' ' 1
##
## R-sq.(adj) =  0.598   Deviance explained = 66.9%
## -REML = 87.321  Scale est. = 136.38    n = 23
```

```
dexa.gam.combined3<-gam(DEXA_fat~skinfold_back + s(skinfold_axilla,
by=weight) ,
method="REML", data=dexa)
options(scipen=999)
summary(dexa.gam.combined3)
```

```
##
## Family: gaussian
## Link function: identity
##
## Formula:
## DEXA_fat ~ skinfold_back + s(skinfold_axilla, by = weight)
##
## Parametric coefficients:
##               Estimate Std. Error t value Pr(>|t|)
## (Intercept)     22.500      9.973   2.256  0.03641 *
## skinfold_back    4.975      1.685   2.953  0.00833 **
## ---
## Signif. codes:  0 '***' 0.001 '**' 0.01 '*' 0.05 '.' 0.1 ' ' 1
##
## Approximate significance of smooth terms:
##                              edf Ref.df     F p-value
## s(skinfold_axilla):weight 2.523  2.857 8.239   0.002 **
## ---
## Signif. codes:  0 '***' 0.001 '**' 0.01 '*' 0.05 '.' 0.1 ' ' 1
##
## R-sq.(adj) =  0.491   Deviance explained = 57.3%
## -REML = 88.872  Scale est. = 172.39    n = 23
```

```
dexa.gam.combined4<-gam(DEXA_fat~skinfold_neck + s(skinfold_axilla,
by=weight) ,
method="REML", data=dexa)
options(scipen=999)
summary(dexa.gam.combined4)
```

```
##
## Family: gaussian
## Link function: identity
##
## Formula:
## DEXA_fat ~ skinfold_neck + s(skinfold_axilla, by = weight)
##
## Parametric coefficients:
##               Estimate Std. Error t value Pr(>|t|)
## (Intercept)    25.1697     6.8136   3.694 0.001704 **
## skinfold_neck   4.2447     0.9769   4.345 0.000406 ***
```

```
## ---
## Signif. codes:  0 '***' 0.001 '**' 0.01 '*' 0.05 '.' 0.1 ' ' 1
##
## Approximate significance of smooth terms:
##                              edf Ref.df     F  p-value
## s(skinfold_axilla):weight 3.329  3.828 10.88 0.000146 ***
## ---
## Signif. codes:  0 '***' 0.001 '**' 0.01 '*' 0.05 '.' 0.1 ' ' 1
##
## R-sq.(adj) =  0.669   Deviance explained = 73.4%
## -REML = 85.938  Scale est. = 112.07    n = 23
```

```
dexa.gam.combined5<-gam(DEXA_fat~skinfold_axilla + s(skinfold_neck,
by=weight) ,
method="REML", data=dexa)
options(scipen=999)
summary(dexa.gam.combined5)
```

```
##
## Family: gaussian
## Link function: identity
##
## Formula:
## DEXA_fat ~ skinfold_axilla + s(skinfold_neck, by = weight)
##
## Parametric coefficients:
##                 Estimate Std. Error t value Pr(>|t|)
## (Intercept)       40.874      9.682   4.222 0.000504 ***
## skinfold_axilla    2.269      1.747   1.299 0.210256
## ---
## Signif. codes:  0 '***' 0.001 '**' 0.01 '*' 0.05 '.' 0.1 ' ' 1
##
## Approximate significance of smooth terms:
##                            edf Ref.df     F p-value
## s(skinfold_neck):weight 2.838  3.235 4.965  0.0101 *
## ---
## Signif. codes:  0 '***' 0.001 '**' 0.01 '*' 0.05 '.' 0.1 ' ' 1
##
## R-sq.(adj) =  0.396   Deviance explained = 50.1%
## -REML = 90.813  Scale est. = 204.78    n = 23
```

```
dexa.gam.combined6<-gam(DEXA_fat~skinfold_axilla + s(skinfold_back,
by=weight) ,
method="REML", data=dexa)
options(scipen=999)
summary(dexa.gam.combined6)
```

```
##
## Family: gaussian
## Link function: identity
##
```

```
## Formula:
## DEXA_fat ~ skinfold_axilla + s(skinfold_back, by = weight)
##
## Parametric coefficients:
##                 Estimate Std. Error t value Pr(>|t|)
## (Intercept)       39.314      8.797   4.469 0.000292 ***
## skinfold_axilla    2.355      1.529   1.540 0.140786
## ---
## Signif. codes:  0 '***' 0.001 '**' 0.01 '*' 0.05 '.' 0.1 ' ' 1
##
## Approximate significance of smooth terms:
##                          edf Ref.df     F p-value
## s(skinfold_back):weight 2.864  3.261 4.827 0.00979 **
## ---
## Signif. codes:  0 '***' 0.001 '**' 0.01 '*' 0.05 '.' 0.1 ' ' 1
##
## R-sq.(adj) =  0.407   Deviance explained = 51.1%
## -REML = 90.796  Scale est. = 201.1     n = 23
```

```
AIC(dexa.gam.combined1)
```

```
## [1] 189.7396
```

```
AIC(dexa.gam.combined2)
```

```
## [1] 185.4146
```

```
AIC(dexa.gam.combined3)
```

```
## [1] 190.3933
```

```
AIC(dexa.gam.combined4)
```

```
## [1] 181.3087
```

```
AIC(dexa.gam.combined5)
```

```
## [1] 194.7142
```

```
AIC(dexa.gam.combined6)
```

```
## [1] 194.3161
```

```
dexa.gam.combined44<-gam(DEXA_fat~skinfold_neck + skinfold_back +
s(skinfold_axilla, by=weight) ,
method="REML", data=dexa)
options(scipen=999)
summary(dexa.gam.combined44)
```

```
##
## Family: gaussian
## Link function: identity
##
```

```
## Formula:
## DEXA_fat ~ skinfold_neck + skinfold_back + s(skinfold_axilla,
##     by = weight)
##
## Parametric coefficients:
##               Estimate Std. Error t value Pr(>|t|)
## (Intercept)   25.48075    9.02675   2.823   0.0118 *
## skinfold_neck  4.28769    1.50476   2.849   0.0112 *
## skinfold_back -0.09638    2.28361  -0.042   0.9668
## ---
## Signif. codes:  0 '***' 0.001 '**' 0.01 '*' 0.05 '.' 0.1 ' ' 1
##
## Approximate significance of smooth terms:
##                              edf Ref.df     F p-value
## s(skinfold_axilla):weight 3.207  3.711 10.48 0.00025 ***
## ---
## Signif. codes:  0 '***' 0.001 '**' 0.01 '*' 0.05 '.' 0.1 ' ' 1
##
## R-sq.(adj) =  0.654   Deviance explained = 73.6%
## -REML = 84.209  Scale est. = 117.4     n = 23
```

```
dexa.gam.combined444<-gam(DEXA_fat~skinfold_neck + s(skinfold_axilla,
by=weight) +s(skinfold_back, by=weight) ,
method="REML", data=dexa)
options(scipen=999)
summary(dexa.gam.combined444)
```

```
##
## Family: gaussian
## Link function: identity
##
## Formula:
## DEXA_fat ~ skinfold_neck + s(skinfold_axilla, by = weight) +
##     s(skinfold_back, by = weight)
##
## Parametric coefficients:
##               Estimate Std. Error t value Pr(>|t|)
## (Intercept)     25.625      7.097   3.611 0.002179 **
## skinfold_neck    4.207      1.005   4.186 0.000628 ***
## ---
## Signif. codes:  0 '***' 0.001 '**' 0.01 '*' 0.05 '.' 0.1 ' ' 1
##
## Approximate significance of smooth terms:
##                              edf Ref.df      F  p-value
## s(skinfold_axilla):weight 3.118  3.509 11.004 0.000207 ***
## s(skinfold_back):weight   1.000  1.000  0.099 0.757154
## ---
## Signif. codes:  0 '***' 0.001 '**' 0.01 '*' 0.05 '.' 0.1 ' ' 1
##
## Rank: 21/22
```

```
## R-sq.(adj) =  0.655   Deviance explained = 73.5%
## -REML = 83.258  Scale est. = 116.93    n = 23
```

```
dexa.gam.combined4.intrinsic<-gam(DEXA_fat~skinfold_neck + s(skinfold_axilla,
by=weight) + age + sex + neutering,
method="REML", data=dexa)
options(scipen=999)
summary(dexa.gam.combined4.intrinsic)
```

```
##
## Family: gaussian
## Link function: identity
##
## Formula:
## DEXA_fat ~ skinfold_neck + s(skinfold_axilla, by = weight) +
##     age + sex + neutering
##
## Parametric coefficients:
##               Estimate Std. Error t value Pr(>|t|)
## (Intercept)    24.6497     8.7296   2.824  0.01295 *
## skinfold_neck   4.1565     1.1901   3.493  0.00332 **
## age             0.1379     0.5993   0.230  0.82117
## sexMale        -4.4878     5.4338  -0.826  0.42196
## neuteringYes    5.0536     8.0463   0.628  0.53951
## ---
## Signif. codes:  0 '***' 0.001 '**' 0.01 '*' 0.05 '.' 0.1 ' ' 1
##
## Approximate significance of smooth terms:
##                               edf Ref.df     F p-value
## s(skinfold_axilla):weight 3.174  3.631 6.524 0.00402 **
## ---
## Signif. codes:  0 '***' 0.001 '**' 0.01 '*' 0.05 '.' 0.1 ' ' 1
##
## R-sq.(adj) =  0.631   Deviance explained = 75.1%
## -REML = 79.428  Scale est. = 125.15    n = 23
```

```
plot(dexa.gam.combined4, xlab = "Objective measurements of skinfold thickness
of the axillar rib (mm)", ylab = "Spline effect: s(Axillar rib, by =
Bodyweight)")
```

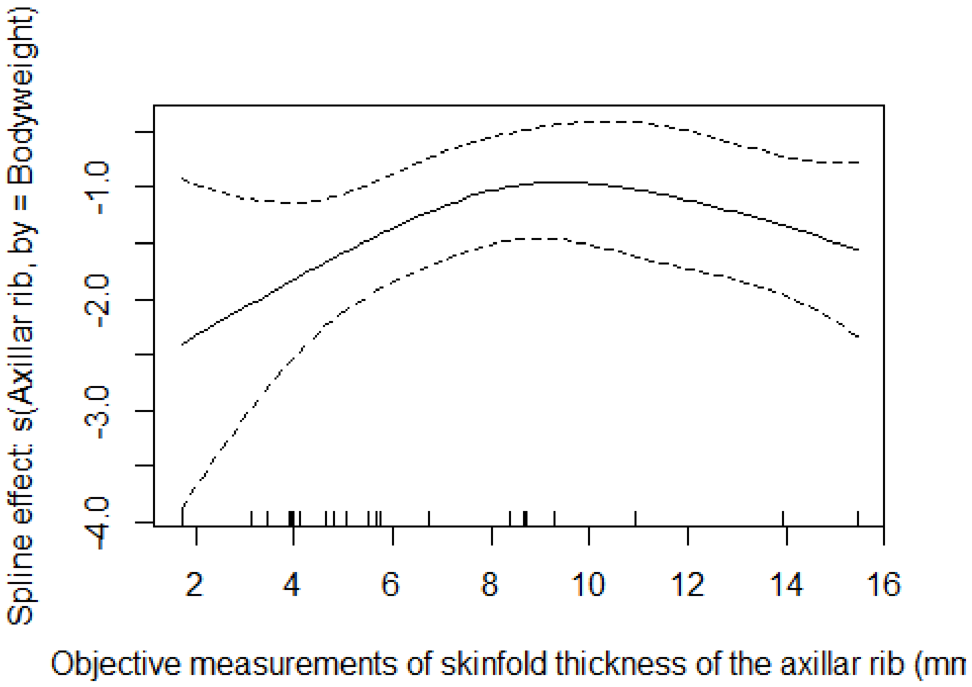


```r
gam.check(dexa.gam.combined4)
```

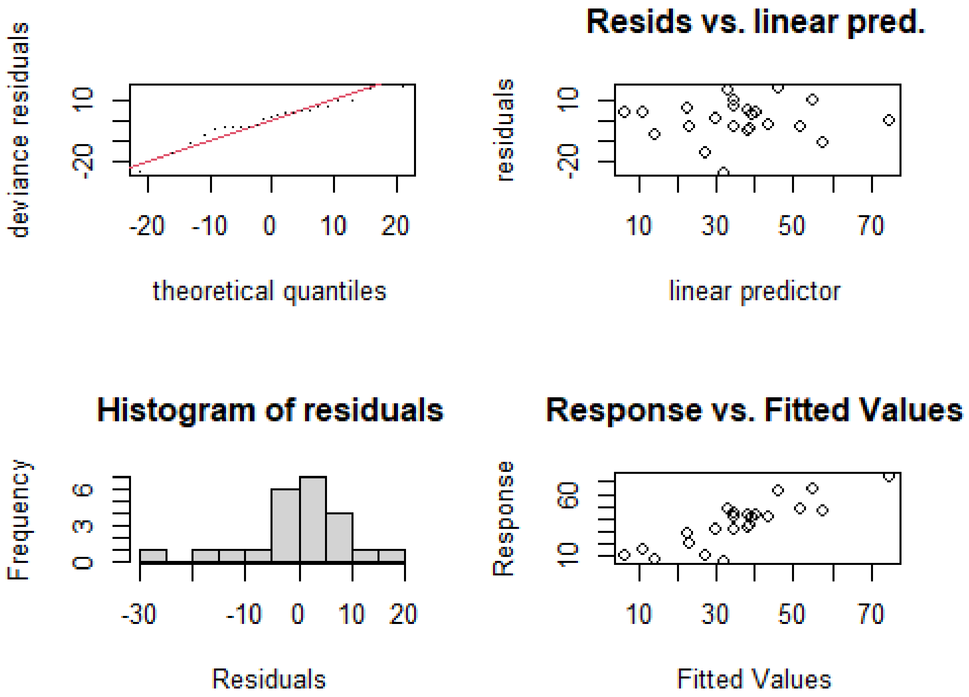


```
##
## Method: REML   Optimizer: outer newton
```

```
## full convergence after 5 iterations.
## Gradient range [-0.000001937868,-0.00000002118356]
## (score 85.93781 & scale 112.0684).
## Hessian positive definite, eigenvalue range [0.5962369,9.549591].
## Model rank =  12 / 12
##
## Basis dimension (k) checking results. Low p-value (k-index<1) may
## indicate that k is too low, especially if edf is close to k'.
##
##                                 k'   edf k-index p-value
## s(skinfold_axilla):weight 10.00  3.33    0.96    0.29
```

```
dexa$pred <- predict(dexa.gam.combined4, newdata = dexa)

dexa %>%
  ggplot(aes(x = pred, y = DEXA_fat, col = as.factor(size))) +
  geom_point() +
  geom_abline(intercept = 0, slope = 1) +
  labs(
    x = "Predicted total body fat percentage (%)",
    y = "Total body fat percentage evaluated by DEXA (%)",
    color = "Size of dogs"  # Optional: renames the color legend
  )
```

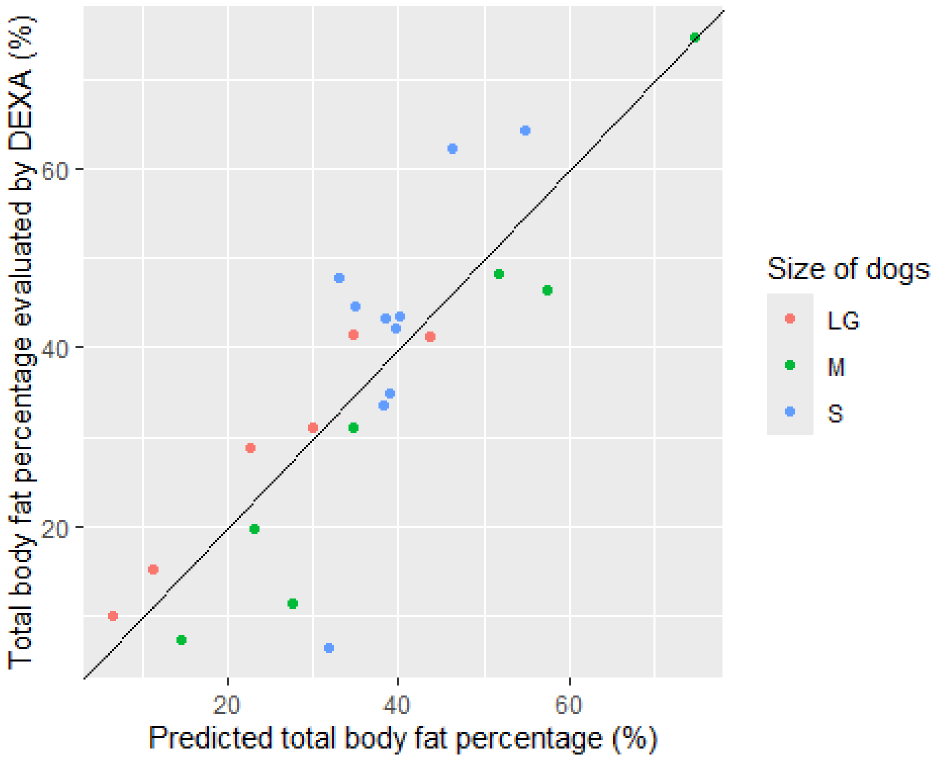


```
n <- nrow(dexa)
predicted <- numeric(n)
observed <- dexa$DEXA_fat
```

```r
bias <- numeric(n)

for (i in 1:n) {
  train <- dexa[-i, ]
  test <- dexa[i, , drop = FALSE]

  gam_model_final <- gam(DEXA_fat~skinfold_neck + s(skinfold_axilla,
by=weight),
                     data = train)

  predicted[i] <- predict(gam_model_final, newdata = test)
  bias[i] <- predicted[i] - test$DEXA_fat
}

rmse <- sqrt(mean((predicted - observed)^2))
mean_bias <- mean(bias)
slope <- coef(lm(observed ~ predicted))[2]

cat("RMSE:", round(rmse, 3), "\n")

## RMSE: 11.327

cat("Mean Bias:", round(mean_bias, 3), "\n")

## Mean Bias: -0.24

cat("Observed vs Predicted Slope:", round(slope, 3), "\n")

## Observed vs Predicted Slope: 0.919

bias_sd <- sd(bias)
bias_se <- bias_sd / sqrt(n)
t_critical <- qt(0.975, df = n - 1)  # 2-tailed 95% CI

ci_lower <- mean_bias - t_critical * bias_se
ci_upper <- mean_bias + t_critical * bias_se

cat("Bias 95% CI:", round(ci_lower, 3), "to", round(ci_upper, 3), "\n")

## Bias 95% CI: -5.247 to 4.767

library(tinytex)

## Warning: package 'tinytex' was built under R version 4.4.3

library (latexpdf)
```
