# Supplementary material for: Objective measurements of skinfold thickness with a caliper show a significant relationship to total body fat percentage in dogs
Source: Front Vet Sci. 2025 Sep 12;12:1656855. doi: 10.3389/fvets.2025.1656855 (PMC12463608; doi:10.3389/fvets.2025.1656855)
Supplement: Supplementary file 4 [file Table_4.pdf]

Supplementary file 4; Descriptive statistics of the Chihuahua dogs.

Supplementary table 1. Descriptive statistics of the four Chihuahua dogs included in the cohort.

**BCS**	**BF%**	**Objective skinfold thickness (mm)**		
		Dorsal neck	Axillar rib	Lumbar back
2	6.4	2.7	1.7	2.3
4	43.5	4.5	3.1	3.5
6	62.3	6.8	3.5	4.9
9	64.2	8.4	4.8	6.8

BCS: Body condition score, BF%: Total body fat percentage, mm: millimeter. Four privately owned newly euthanized dogs were assessed for BCS by the 9-point BCS scale (Laflamme 1997), evaluated for BF% by Dual-Energy X-ray Absorptiometry (DEXA) and by objective measurements for skinfold thickness at the location of the dorsal neck, the axillar rib and the lumbar back by a caliper. Objective measurements of skinfold thickness are here shown as the mean of the triplicate for both observers in millimeter (mm).
